# Electric field stimulation directs target-specific axon regeneration and partial restoration of vision after optic nerve crush injury

**DOI:** 10.1371/journal.pone.0315562

**Published:** 2025-01-09

**Authors:** Timothy Kim, Ege Iseri, Micalla G. Peng, Sasha Medvidovic, Timothy Silliman, Pooyan Pahlavan, Gengle Niu, Connie Huang, Anahit Simonyan, Javad Pahnahad, Petcy Yao, Phillip Lam, Vahini Garimella, Mahnaz Shahidi, Michael S. Bienkowski, Darrin J. Lee, Biju Thomas, Gianluca Lazzi, Kimberly K. Gokoffski

**Affiliations:** 1 Department of Ophthalmology, Keck School of Medicine, USC Roski Eye Institute, University of Southern California, Los Angeles, California, United States of America; 2 Department of Biomedical Engineering, Viterbi School of Engineering, University of Southern California, Los Angeles, California, United States of America; 3 Institute for Technology and Medical Systems (ITEMS), Keck School of Medicine, University of Southern California, Los Angeles, California, United States of America; 4 Department of Electrical and Computer Engineering, Viterbi School of Engineering, University of Southern California, Los Angeles, California, United States of America; 5 Boston Scientific Neuromodulation, Valencia, California, United States of America; 6 Johnson & Johnson, Irvine, California, United States of America; 7 Stevens Neuroimaging and Informatics Institute, Keck School of Medicine, University of Southern California, Los Angeles, California, United States of America; 8 Department of Neurosurgery, Keck School of Medicine, University of Southern California, Los Angeles, California, United States of America; Roskamp Institute, UNITED STATES OF AMERICA

## Abstract

Failure of central nervous system (CNS) axons to regenerate after injury results in permanent disability. Several molecular neuro-protective and neuro-regenerative strategies have been proposed as potential treatments but do not provide the directional cues needed to direct target-specific axon regeneration. Here, we demonstrate that applying an external guidance cue in the form of electric field stimulation to adult rats after optic nerve crush injury was effective at directing long-distance, target-specific retinal ganglion cell (RGC) axon regeneration to native targets in the diencephalon. Stimulation was performed with asymmetric charged-balanced (ACB) waveforms that are safer than direct current and more effective than traditional, symmetric biphasic waveforms. In addition to partial anatomical restoration, ACB waveforms conferred partial restoration of visual function as measured by pattern electroretinogram recordings and local field potential recordings in the superior colliculus—and did so without the need for genetic manipulation. Our work suggests that exogenous electric field application can override cell-intrinsic and cell-extrinsic barriers to axon regeneration, and that electrical stimulation performed with specific ACB waveforms may be an effective strategy for directing anatomical and functional restoration after CNS injury.

## Introduction

Failure of neurons to regenerate after injury in the central nervous system (CNS) is a consequence of diminished intrinsic growth capacity of adult neurons (cell-intrinsic barriers) and inhibitory signals in the extracellular environment (cell-extrinsic barriers) [1, 2]. To date, most approaches to drive long-distance axon regeneration focus on overcoming cell-intrinsic barriers—via upregulation of dormant signaling pathways that were active during development to revert cells to a developmental or growth state. In the case of the optic nerve, a screen for evolutionarily conserved signaling pathways that control cellular growth identified mTOR (mammalian target of rapamycin) suppression as a major barrier to retinal ganglion cell (RGC) axon regeneration in mice. Deletion of PTEN (phosphatase and tensin homolog), a negative regulator of mTOR, led to long-distance RGC axon regeneration after optic nerve crush injury [3]. Since then, RGC axon regeneration has been reported with genetic modulation of other signaling pathways including SOCS3 (suppressor of cytokine signaling 3) and the Yamanaka genes [4, 5]. Increased gains were reported when these neuro-regenerative strategies were combined with overexpression of neurotrophic factors such as CNTF (ciliary neurotrophic factor), erythropoietin, intraocular injection of pro-inflammatory molecules like zymosan, or chelation of zinc [6–10].

Although these strategies have significantly advanced the field of optic nerve regeneration, they do not address the need to provide growing axons with directional cues. As a result, regenerating RGC axons were found to stall at the optic chiasm in some cases [3, 5]. In PTEN/SOCS3 knock out (KO) mice and PTEN KO/zymosan/cAMP-treated mice, 10–20% of axons exhibited premature branching and 40% of axons regenerated aberrantly, making U-turns and extending back towards the eye or growing into the contralateral optic nerve [11]. Of those animals that demonstrated retrochiasmal growth, RGC axons projected to bilateral suprachiasmatic nuclei (SCN), but few RGC axons were found to project to distal structures including the superior colliculus (SC) or lateral geniculate nucleus (LGN) [11, 12].

Restoration of function in the CNS requires targeted axon regeneration. However, development of strategies that can recapitulate the guidance cues that directed axon growth during development have been limited by the need to not only express these cues in spatial but also temporal gradients [13]. For example, in the developing Drosophila CNS, axon decussation requires neurons to exchange FasII (Fasciclin II) surface receptors for FasI. Once decussation is complete, neurons must then switch back to express only FasII [14]. Successful recapitulation of these gradients using current molecular techniques has been limited.

Electric fields (EFs) may serve as a unique solution to this problem. Electric potentials are naturally generated in the body, and EFs have been shown to direct tissue pattern during development and wound healing after injury [15]. We and other groups have shown that EFs confer both pro-survival and pro-regenerative benefits on RGCs where immediate application of EFs after optic nerve transection promoted 1.5-fold more RGC survival over controls [16]. EF stimulation enhances RGC axon growth in response to neurotrophic factors including brain-derived neurotrophic factor (BDNF) [17]. Notably, we have demonstrated that EFs not only promote RGC axon growth but also control the direction of growing axons [18]. RGC axons acutely respond to changes in EF orientation by turning and redirecting their growth cones towards the cathode, the electrode with the more negative potential, in vitro. We now show that in vivo EF stimulation can direct full-length, target-specific RGC axon regeneration in adult rodents after optic nerve crush injury, without evidence of aberrant regeneration. In addition to anatomical restoration, EF stimulation partially restored electrophysiologic function and did so without the need for concurrent genetic manipulation. Our findings illustrate the potential of exogenous EF application to provide the missing link for directing target-specific regeneration in the mature CNS.

## Materials and methods

### Surgical implantation of electrodes and optic nerve crush

**Fig 1:**
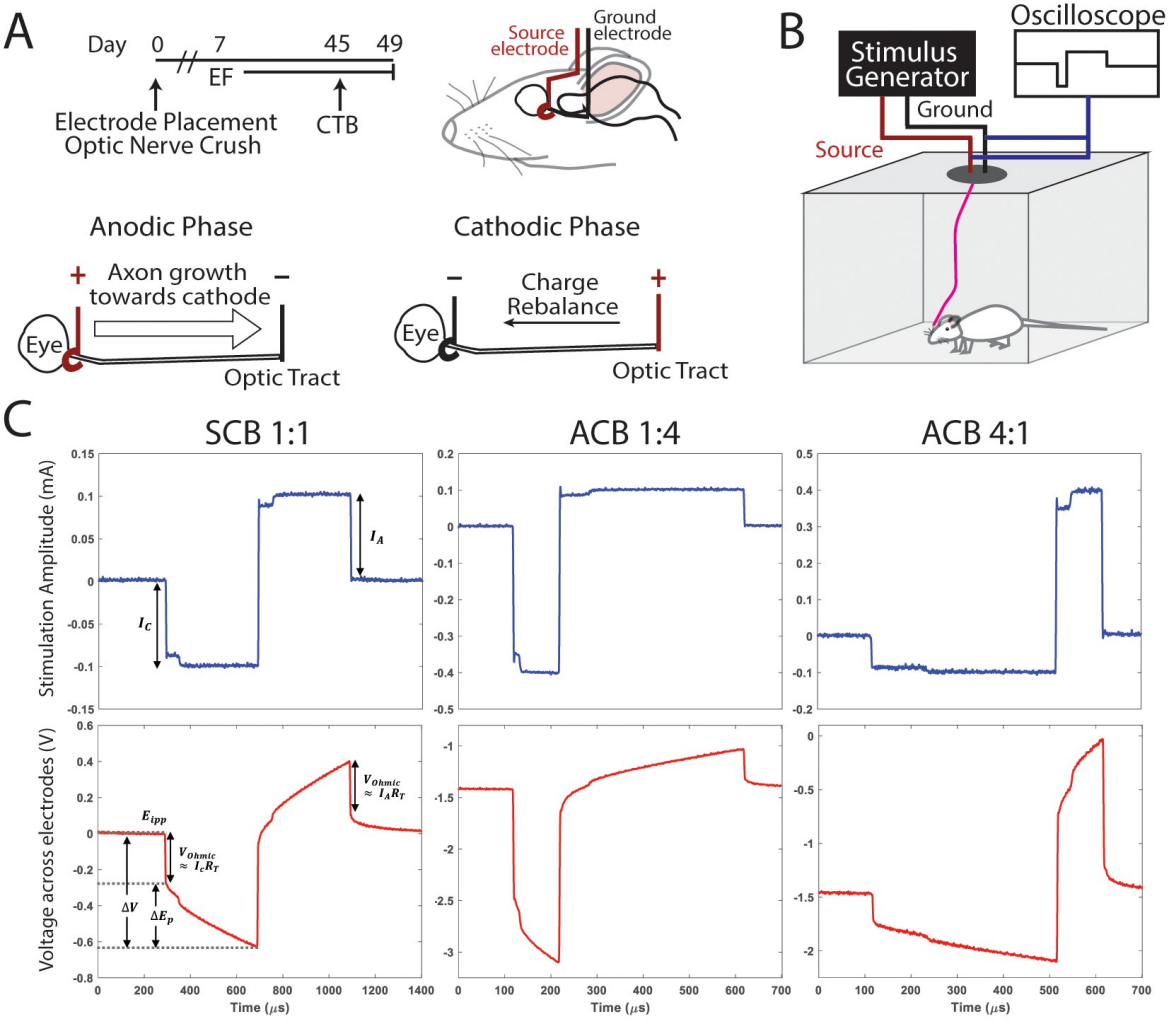
Schematic of experimental timeline, stimulation system, and stimulation waveforms. (A) Adult Long-Evans rats underwent optic nerve crush and concurrent electrode placement. Five to seven days later, optic nerves were stimulated with various waveforms (C) for 5 hours/day every weekday for 6 weeks. Schematic of orientation of electric field (EF) gradient during anodic and cathodic phases. Three to four days before euthanasia, rats underwent intravitreal injection with cholera toxin (CTB)-647. (B) Rats were attached to a stimulation generator via a tether on a ball joint, allowing for continuous stimulation in an unanesthetized, mobile rat. Continuous waveform monitoring with an oscilloscope was performed to assure that rats were being stimulated appropriately. (C) The current stimulation pulses of various waveforms recorded across a 1 kΩ resistor connected in series to the return electrode (top row) and the resulting voltage transient measured across the platinum needle electrodes (bottom row). *E*_*ipp*_ is the electrode potential at the onset of the current pulse (interphase), *Δ*V is the total polarization of the electrode during the stimulation phase, *V*_*Ohmic*_ is the near-instantaneous potential change at the onset/termination of the current pulse following the characteristics of a purely resistive load representing the stimulated tissue (*R*_*T*_), and *ΔE*_*p*_ is the steady-state potential following the characteristics of a reactive (capacitive) load. *E*_*ipp*_ for the SCB 1:1 was centered at zero for better visualization of the values. SCB, symmetric charge-balanced; ACB, asymmetric charge-balanced. Numbers represent the relative cathodic:anodic pulse-width ratios.

The use of animals was in accordance with the ARRIVE guideline, the Association for Research in Vision and Ophthalmology (ARVO) Statement on the use of animals for research and was approved by the Institutional Animal Care and Use Committee at the University of Southern California [19]. After sedation with isoflurane, adult male Long-Evans rats (300–500 g; Charles River; Wilmington, MA) underwent a midsagittal scalp incision to expose the bregma and lambda followed by a left lateral canthotomy and cantholysis to expose the left optic nerve. Blunt dissection was performed under the frontalis muscle to create a tunnel between the orbit and the scalp incision. A 25 mm long platinum (Pt) “J-shaped” source electrode (250 *μ*m diameter; P1 Technologies; Boerne, TX) with 5 mm of its insulation coating removed was tunneled from the scalp incision into the orbit, under the lateral rectus, and then wrapped around the left optic nerve at the base of the globe. A straight 16 mm long Pt ground electrode with 1 mm of its insulation coating removed was placed stereotactically (2.43 M-L, -1.00 A-P, -9.65 D-V at 10°; Stoelting 51900; Wood Dale, IL) into the contralateral optic tract (Fig 1).

Both source and ground electrodes were made of Pt [20, 21]. The source electrode was made into a “J-shape” to maximize contact with the optic nerve, which was assumed to move and distort with eye movement. During the same surgery, rats then underwent optic nerve crush injury for 10 seconds using cross-action #5 Dumont forceps (World Precision Instruments; Sarasota, FL). The electrodes were then secured into place with dental acrylic (Patterson Dental; El Segundo, CA) and a plastic pinwheel pedestal (P1 Technologies; Boerne, TX). The lateral canthus was repaired and dexamethasone/neomycin sulfate/polymyxin B ophthalmic ointment (MWI Veterinary Supply 044523; Boise, ID) applied to the eye. To minimize post-operative pain, rats underwent subcutaneous injection of extended-release buprenorphine (0.65 mg/kg, Ethiqa XR, MWI Animal Health, Boise, ID) once immediately before surgery and given oral meloxicam (1–2 mg/kg) once daily for 3 days after surgery.

### Electrode connection and stimulation parameters

Electrical stimulation was delivered along the optic nerve using a two-electrode configuration, where the source electrode was placed around the left optic nerve and the return electrode (ground) within the contralateral optic tract (Fig 1A). A current-controlled stimulus generator (STG 4008, Multi-Channel Systems, Harvard Bioscience, Inc.) was connected to the source and ground electrodes via a wire tether that was attached to a commutator with a ball joint (P1 instruments; Fig 1B). This allowed the unanesthetized rat to roam freely within the cage during stimulation. Constant current mode was selected to ensure the delivery of a stable current between the electrodes independent of changes in the electrode or tissue properties that might affect impedance during the stimulation period. This configuration has been shown to generate voltage gradients, and thus EFs, along the optic nerve [21].

Three custom waveforms were used in the trials: 1) symmetric biphasic, 2) asymmetric biphasic, anodically-driven, and 3) asymmetric biphasic, cathodically-driven, all at 50% duty cycle (Fig 1C). The waveform parameters are provided in S1 Table. Each custom waveform was programmed using the MC Stimulus II software (Harvard Biosciences, Germany). On day 1 (one week after surgery), stimulation was initiated at 100% amplitude and 5% duty cycle. Duty cycle was increased every hour as tolerated until 50% duty cycle was reached. Rats were considered to be intolerant to the stimulation if they displayed physical behaviors such as eyelid fasciculations, or ceased expected behaviors such as grooming, sleeping, or exploration of their cage.

To ensure that rats were receiving the correct stimulation, waveforms were monitored continuously with an oscilloscope. As the compliance voltage of this stimulation system was an order of magnitude larger than the voltage necessary to pass the target current through the impedance of the electrode-tissue interface, the rats were essentially guaranteed to receive the full input current for the entire duration of the experiment. The stability of the current delivery was verified by connecting a 1 kΩ series resistor to the ground terminal and measuring the voltage drop across it. The voltage generated between the electrodes was periodically measured to assess for changes in the stimulated tissue or electrode-tissue interface over time. Once animals reached their endpoint, they were euthanized with CO_2_ followed by thoracotomy and cardiac perfusion.

### 3D computational model of optic nerve and neuronal response to electrical stimulation

The Admittance Method (AM) developed by our group was used to predict the voltage distribution along the rat optic nerve and the retina by our stimulation setup [22–25]. Briefly, a high-resolution 3D image of the rodent head and orbital structures was obtained using computed tomography performed on a CD1 adult mouse [26]. This image was then discretized and imported into the AM computational platform. The size of the head, and the length and angle of the optic nerve were then adjusted according to the measurements taken in an adult Long-Evans rat. This generated a model consisting of 912, 900, and 504 voxels in the x, y, and z directions respectively, with each voxel being a cube with a length of 83 *μ*m. Resistivity of different tissues within the rat head was assigned according to prior work (S2 Table) [27–29].

The optic nerve was represented as a cylinder starting from behind the eye and extending to the optic chiasm with a diameter of 500 *μ*m. The optic nerve was modeled to be surrounded by a thin layer of cerebrospinal fluid (CSF) during its course within the orbit, surrounded by a large layer of fat, a thin discrete layer of muscle to represent the recti muscles, and finally enclosed in bone. Electrode size and position were modeled according to the surgical positions described above. To predict how individual RGCs may respond to stimulation, we generated a realistic morphological model of a RGC cell as a .swc file and assigned membrane properties specific to its subtype [30]. The extracellular voltage mapping was interpolated onto the RGC morphologies in the NEURON simulation environment to calculate the change in their membrane potentials at every time step as previously described [31–33]. We modeled the extracellular potential that the RGCs are exposed to depending on these waveform characteristics.

### Visual function assessment

#### 1) Pattern electroretinogram recordings

Pattern electroretinogram (PERG) recordings were performed with a UTAS Visual Electrodiagnostic System (LKC, Gaithersburg, MD), using DTL-plus electrodes (M019258 and M014764, Jorvec, Miami, FL). Rats were sedated with inhaled isoflurane followed by an intraperitoneal injection of a ketamine/xylazine cocktail (5/37.5 mg/kg, respectively). Rats were then placed on the system’s thermostatically controlled heating pad and positioned such that the projection of the pupil was aligned to the center of the pattern monitor 20 cm away. The eyes were not dilated, in keeping with prior publications [34]. The ground and reference electrodes were superficially inserted into the base of the tail and snout, respectively, while recording electrodes were placed on the corneal surface with careful attention to not block the pupil. The cornea was kept moist with lubricating eye drops. Impedance was checked three times prior to running the experiment. Rats were evaluated without dark adaptation to limit the possibility of direct photoreceptor-mediated evoked responses [35].

The pattern stimuli were displayed on an RGB monitor (model Dell M933S; Dell, Inc., Round Rock, TX). Stimuli consisted of 0.05 cyl/deg horizontal black and white bars reversing at 1 Hz, presented at 100% contrast and an average ambient room lighting mean luminance of 100 cd/*m*^2^. A range of 992 to 1488 signals were obtained and averaged per rat at each timepoint to isolate the response from background noise. The amplitude was measured from the P50 peak to the N95 trough. The peak time was measured as the time to the peak of the positive response. Normalized amplitudes are reported as left eye amplitude divided by the corresponding right eye amplitude. A two-way ANOVA with Tukey’s test for multiple comparisons test was performed on the normalized amplitudes. Responses not within four standard deviations of the mean amplitude of the group were regarded as artifact and discarded [34].

#### 2) Local field potential recordings

Stereotactically-guided local field potential (LFP) recordings in the superior colliculus (SC) were performed as previously described [36]. After dark-adaptation overnight, rats were sedated with an intraperitoneal injection of xylazine/ketamine (5/37.5 mg/kg respectively) and maintained on 1%-3% inhaled sevoflurane. Pupils were dilated with 0.5% tropicamide and 2.5% phenylephrine. The scalp was carefully separated from the dental acrylic to expose the orbital electrode. The electrode was then cut and the dental acrylic along with the intracranial electrode removed. Rats were placed in a stereotactic apparatus (David Kopf Instruments, Tijunga, CA), and a right parietal craniotomy was performed with a handheld drill (Dremel, Walnut Ridge, AR). A small amount of cortex overlying the right SC was then aspirated to directly visualize the SC. The stereotactic apparatus was referenced from the lambda suture and used to guide placement of a single custom-made tungsten recording electrode within the right SC. The reference electrode was placed near the exposed scalp, and the ground electrode was placed subcutaneously in the tail region. Electrical response to a full-field flash of 1300 cd/*m*^2^ (Grass model PS 33 Photic stimulator, W. Warwick, RI) was recorded using the Powerlab data acquisition system (ADInstruments, Mountain View, CA). Recordings were performed from 28–30 SC locations per animal (200–400 *μ*m apart) covering the fullest visible extent of the SC. Based on the SC activity, 3–10 trials were performed at each recording site. Stereotactic coordinates of the electrode penetrations were recorded at each site. Light stimulus was time locked with the recording device. At the termination of the experiment, animals were euthanized with an overdose of intraperitoneal sodium pentobarbatol/phenytoin (150 mg/kg; Euthasol, MWI Veterinary Supply, Orlando, FL) followed by cardiac perfusion.

LFP recordings were analyzed using LabChart 8 Reader (ADInstruments, Mountain View, CA). Visual responses to light were defined as a clear, prolonged (>20 msec) increase (at least two-fold) in the light-evoked electrical activity above background activity (determined using the 100 msec of activity recorded prior to light stimulus). Recordings were analyzed for (1) percentage of visually responsive sites, (2) response onset latency, defined as time from stimulus to the earliest point of visual response, and (3) peak response amplitude, defined as the largest excursion peak to peak during the visual response [36, 37]. The response onset latencies and peak response amplitudes were averaged across all trials from each recording location.

#### 3) Pupillary response

To assess direct pupillary light reflex, rats underwent tarsorrhaphy of the uninjured eye to prevent consensual pupillary constriction. After dark adaptation for at least 1 hour, the rats were sedated under inhaled isoflurane and the injured eye was gently opened with manual traction. Photic stimulus was provided by an LED lamp (GearLight, Walpole, MA) measured to deliver a high intensity light of 1500 lux at 20 cm from the eye. Pupillary response was video recorded at 3x magnification using an Apple iPhone 13 Pro for at least 45 seconds. The rate and extent of pupillary constriction were quantified on ImageJ by measuring the change in ratio of pupil diameter to limbus diameter at 0, 10, and 30 seconds after start of photic stimulation [38, 39]. Measurements were performed in triplicates and averaged. During testing, rats were neither awake nor handheld to avoid the possibility of sympathetically-driven pupillary dilation that could interfere with measurements.

#### 4) Optokinetic reflex testing

Optokinetic reflex (OKR) testing was performed similar to Ahmed et al [40]. All rats were dark-adapted for at least 15 minutes prior to the experiment. Rats were placed on a platform positioned 10 inches above the ground and 4.5 inches away from two Apple iPad tablets (5th generation, 9.7-in display) positioned lengthwise against each other at a 155° angle to create a seamless overlap of both screens. The OKN Stripes Visualization Web Application program was then opened and set to the lowest spatial frequency of 0.08 c/d. The spatial frequency was gradually increased from 7 to 15 stripes in increments of 2 stripes while maintaining a constant speed of 20 seconds across the screen. Head tracking was monitored for a positive response with either right or leftward rotating lines, respectively. A response was considered positive if the rat was observed to perform a head tracking movement in the same direction of the stripes. The rat was deemed non-responsive to the visual stimuli when no tracking movement was detected for at least 5 seconds on either side over at least two trials. Responses were recorded as able or unable to track for each eye. OKR testing was conducted weekly.

#### 5) Visual cliff test

The visual cliff avoidance test was performed as previously described [41]. A clear, open-top plexiglass box (Conduct Science, Skokie, IL) measuring 82 x 82 cm was placed on a 3-foot-tall table with half of the box protruding. A black-and-white checkerboard pattern was displayed directly underneath the box on the counter (shallow side) and 3 feet below the protruding portion of the box on the floor (deep side). A 25 x 15 x 7.5 cm platform was stationed in the center of the box bisecting the shallow and deep sides. To assure that responses were a result of regeneration in the lesioned eye, tarsorrhaphy was performed on the uninjured right eye. The following day, rats were placed on the platform and scored as follows (1) failure to dismount, which was considered as “failed depth perception” (2) correct dismount to shallow side, which was considered “successful depth perception”, or (3) incorrect dismount to deep side, which was considered “failed depth perception.” Rats were observed for 5 minutes, and each trial was video recorded. Only one trial was performed to avoid the confounding influence of memory effect [41].

### Anatomical assessments

#### 1) RGC axon labeling and quantification

Three to four days before euthanasia, cholera-toxin subunit-B (CTB) conjugated to Alexa-Fluor-647 (CTB-647; C34778, Thermofisher, Waltham, MA) was injected intravitreally to label RGC axons. Briefly, rats were sedated with inhaled isoflurane (2–4%). Pupils were dilated with 0.5% tropicamide and 2.5% phenylephrine. Using a 30G syringe, an anterior chamber tap was performed to lower intraocular pressure. The same needle was used to generate a tunnel through the sclera at the level of the ora serrata. Through this hole, 2 *μ*L of 2 mg/ml CTB-647 was injected intravitreally using a 33G Hamilton syringe (NANOFIL10, World Precision Instruments, Sarasota, FL). Visible filling of the vitreous was confirmed directly under a surgical microscope. Similarly, to label RGC axons, a subset of animals treated with ACB 1:4 underwent intravitreal injection of AAV2-CAG-GFP (6*x*10^9^GC/*μ*l; VectorBiolabs, Malvern, PA) two weeks before crush injury.

Rats were euthanized and subsequently perfused with PBS followed by 4% paraformaldehyde (PFA). Optic nerves were dissected out whole and cleared using a modified version of a previously described protocol [42]. Briefly, the nerves were rinsed twice in PBS for 5 minutes then dehydrated in ascending ethanol series (50%, 80%, 95%) for 1 hour each under gentle agitation and in 100% ethanol overnight at room temperature (RT). The nerves were further dehydrated in hexane (EM-131 HX0304–6, VWR, Radnor, PA) overnight at RT, then transferred into a clearing solution (BABB) of 1 part benzyl alcohol (103514–446, VWR, Radnor, PA) in 2 parts benzyl benzoate (200000–094, VWR, Radnor, PA) and left overnight at RT under agitation. Optic nerves were then placed in a 35 mm glass bottom dish (MatTek, P35G-0–20-C, Ashland, MA) in BABB solution sealed with a glass coverslip (89167–110, VWR, Radnor, PA). A confocal laser scanning microscope (LSM 800, Zeiss, Germany) was used to image the optic nerves at 20x magnification in longitudinal optical sections of 20 *μ*m increments, with tiling set to 512 px x 512 px, 16-bit, and 221 *μ*m pinhole. All optical sections per nerve were saved in a single .czi file for analysis.

Axon quantification was performed as previously described [42]. Briefly, a semi-automated method was used. The fluorescent intensity of individual pixels from CTB labeling served as a surrogate for axon density in specific regions of interests (ROIs) drawn perpendicular to the long axis of the nerve. Optic nerve images were opened in ImageJ/FIJI at the highest resolution and the nerves straightened. ROIs were designated at the pre-crush site (ie, the region of the optic nerve between the globe and crush site), and up to 2000 *μ*m past the crush site in 250 *μ*m increments (collectively, the post-crush ROIs). Threshold pixel intensity was determined to exclude background noise from subsequent analysis using AxonQuantifier, a freely available ImageJ plugin. AxonQuantifier was run to quantify the fluorescence intensities of each post-crush ROI throughout its longitudinal optical sections and normalized to the fluorescence intensity of the pre-crush ROI, and thereby determine the percent of RGC axon density at specified distances from the crush site.

#### 2) Immunohistochemistry and RGC quantification

Eyes were immersed in 4% PFA for 45 minutes at RT then transferred to 0.3% Triton X-100 (VWR, Radnow, PA) and stored at 4°C overnight. The retinas were dissected and cut in four corners and then washed for 5 minutes three times with 0.3% Triton X-100 followed by blocking with 3% horse serum (VWR, Radnow, PA, USA) and 1% Triton X-100 for 30 minutes at RT. The retinas were then incubated with rabbit anti-RBPMS (RNA Binding Protein Multiple Splice; 1:500, GenTex, Irvine, CA) polyclonal antibody for 5 days at 4°C [43]. The retina was washed repeatedly and then incubated with Alexa Fluor 488-conjugated goat anti-rabbit IgG (1:200; Jackson ImmunoResearch, West Grove, PA) secondary antibody overnight at 4°C. After repeating the washing step, retinas were flat mounted on glass slides and sealed with 1.5-mm coverslips with antifade mounting medium (ProLong Diamond; Life Technologies, Eugene, OR) and dried overnight before imaging.

An upright fluorescence microscope (Axio Observer 7, Zeiss, Germany) was used to obtain images using a 20X objective. Images were captured with a charge-coupled device camera and the SimplePCI 5.3 imaging system (Hamamatsu Photonics, Hamamatus City, Japan). The number of RBPMS-positive cells and area of the image were quantified. At least three images from the center, middle, and peripheral area of each quadrant (minimum 12 per retina) were analyzed per rat, totaling at least 1.7 *mm*^2^ of retina per animal.

#### 3) Brain histology

Brains were fixed in 4% PFA, cryoprotected in graded sucrose series (10%, 20%, 30%), and then embedded in OCT (Sakura Finetek, Torrance, CA) before storage at -80°C. Brains were cryosectioned (QS12 Cryostat, Avantik, Pine Brook, NJ) at 25 *μ*m thickness in the coronal plane. Sections were rinsed in PBS, counterstained with 0.15 *μ*g/ml Hoechst (33342, Enzo Life Sciences, Farmingdale, NY) and rinsed again in PBS prior to mounting. The samples were then sealed with 1.5-mm coverslips and antifade mounting medium (ProLong Diamond; Life Technologies, Eugene, OR) and dried overnight. Subcortical targets of the retinofugal pathway were imaged with a Andor Dragonfly spinning disk confocal microscope (Oxford Instruments, United Kingdom) equipped with a 25x Nikon silicon oil objective. All images are MIP imaged with 1 *μ*m Z step through the tissue section.

### Calcium imaging experiments

C57BL/6J-Tg(Thy1-GCaMP6f)GP5.17Dkim/J mice were obtained from Jackson Laboratory (Bar Harbor, ME) [44]. Globes were enucleated from postnatal day (P) 10 to 21 pups in oxygenated room-temperature AMES medium (A1372–25, US Biological, Swampscott, MA) and retinal flat mounts were dissected onto Omnipore 0.1 *μ*m PTFE membranes (Millipore, Cork, Ireland) RGC layer facing up. The membrane was then inverted and placed onto a glass bottom well made in the lab using Anycubic standard resin and 22 mm x 22 mm glass cover slips from Globe Scientific, which was continuously perfused with oxygenated-AMES media. Two tungsten plate electrodes were placed adjacent to opposite ends of the whole-mount retina, 8.5 mm apart, and used to create a consistent EF throughout the well. A -10 V:+2.5 V ACB 1:4 waveform with a 66% duty cycle was applied to the retina in 100 pulse bursts. An SCB -2.5 V:2.5 V 1:1 waveform was also applied to give a comparative baseline of activation in a given field of view. Images were taken with an Andor Ixon Ultra 897 camera, 20x objective. Images were acquired every tenth of a second for a full minute, and a set pattern of stimulation was timed to start alongside the recording, with the first stimulation occurring 7 secs into the recording. Total overall activation in tissue adjacent to the cathode followed by the anode (after polarity was inverted) was quantified using the total mean grey value of the field of view. Two minutes was allowed to lapse between polarity switches and before recordings were resumed. The mean grey value of the field of view of the video at each time point was taken. The mean grey value in a region of interest containing only inactive cells was subtracted from the total mean grey value of the field of view to remove artifacts. Next, the average of the first 50 datapoints were subtracted to normalize all the data around a baseline of zero. Finally, for each recording the average was taken of the 1:1 pulse peaks and the 1:4 pulse peaks and these averages were put into a ratio to compare relative anodic 1:4 stimulation against 1:4 cathodic stimulation.

### Statistical analysis

Results are reported as either standard deviation (STD) or standard error of the mean (SEM) as specified in the text. Significant differences were determined using one-way or two-way analysis of variance [45] followed by Tukey’s post hoc test for multiple comparisons using GraphPad Prism 7 (San Diego, CA). P < 0.05 was considered to indicate a statistically significant difference. Investigators were masked to experimental conditions when analyzing the data.

## Results

### Waveform design

Adult rats underwent optic nerve crush injury concurrently with electrode placement (Fig 1A). However, to mimic clinical scenarios of acute optic nerve injury, electrical stimulation was not initiated until 5 to 7 days after crush injury (Fig 1B). Rats were stimulated with an asymmetric charge balanced (ACB 1:4; cathodic:anodic phase-width ratio) waveform, a waveform in which the duration of the anodic phase was 4 times longer than that of the cathodic phase (Fig 1C). To maintain charge balance, the amplitude of the cathodic phase was set to be four times higher than that of the anodic phase. Given that prior work showed that pulse width exerts a stronger influence on directing RGC axon growth than pulse amplitude [46], we chose to test this waveform as the anodic pulse of ACB 1:4 should act as the working phase, driving axons to grow towards the brain, while the cathodic phase acts as the charge rebalancing phase. Other rats were treated with either a symmetric charge-balanced waveform (SCB 1:1) or ACB 4:1 (the inverse of ACB 1:4; Fig 1C). Rats were continuously stimulated for 5 hours each weekday for 6 weeks (30 days of total stimulation).

In vivo measurements of total injected charge across a 1 kΩ resistor indicated that our biphasic waveforms are charge-balanced (Fig 1C). The cathodic to anodic area was 0.98, 0.98, and 1.01 for ACB 1:4, SCB 1:1, and ACB 4:1 stimulated rats, respectively.

### Electrical stimulation of the optic nerve

To assess the stability of electrical stimulation on the optic nerve, we measured the change in tissue resistance over time. We measured the near-instantaneous voltage change at the onset and termination of the current pulse to determine the approximate voltage drop across the optic nerve. We then calculated the resistance value from these measurements using a simplified version of the equation presented in Cogan et al. for calculating the voltage drop (*V*_*a*_) across the electrolyte (tissue) and estimating its resistance (*R*_*T*_) in the Ohmic relationship *V*_*a*_ = *IR*_*T*_ (Fig 1C) [47]. We used the same method for untreated rats by applying a transient 1:1 SCB stimulation pulse at 25% amplitude and 10% duty cycle to minimize any unwanted stimulation effect. We found the average change in the approximated tissue resistance over 6 weeks to be +0.7% +/- 5.4%, -11.5% +/- 6.1%, +4.9% +/- 7.6%, and -5.7 +/- 11.4% for untreated, SCB 1:1, ACB 1:4, and ACB 4:1 treated rats, respectively. We detected no significant difference between untreated animals and animals stimulated with any of these waveforms (N = 4, one-way ANOVA with Tukey’s multiple comparisons test). The minimal change in tissue resistance over time suggests that our system was able to generate a consistent EF along the optic nerve over the 6-week course.

### Biphasic stimulation with ACB 1:4 directs full-length regeneration of crushed RGC axons

**Fig 2:**
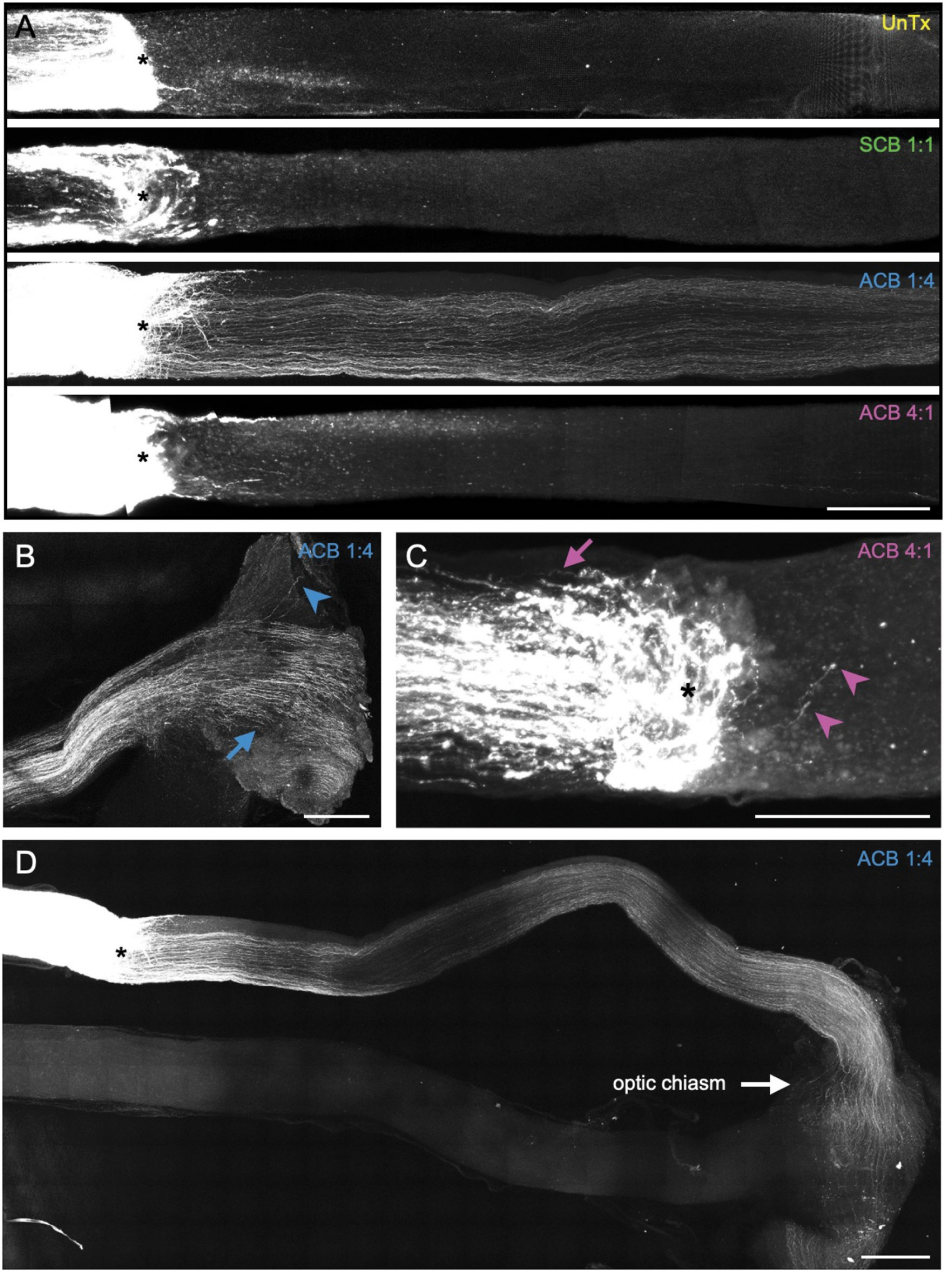
Biphasic stimulation with asymmetric charge-balanced (ACB) 1:4 waveforms directs full-length regeneration of crushed retinal ganglion cell (RGC) axons. (A) Optic nerves underwent crush injury followed by 6 weeks of stimulation with various biphasic waveforms and cholera toxin B (CTB) labeling of RGC axons. Regenerated axons observed past the crush site (asterisk) with ACB 1:4 treatment. (B) Decussating (blue arrow) and non-decussating (blue arrowhead) RGC axons seen in the optic chiasm of an ACB 1:4-treated animal. (C) Pre- and post-crush RGC axons (pink arrow and arrowheads, respectively) observed at the crush site (asterisk) projecting back towards the eye in an ACB 4:1-treated animal. (D) Whole optic nerve of an ACB 1:4-treated animal demonstrating long-distance RGC axon regeneration past the crush site (asterisk) and through the optic chiasm. Scale bars, 250 *μ*m. SCB, symmetric charge-balanced stimulation.

In unstimulated animals, only a few axons were seen extending past the crush site at 250 *μ*m and almost no axons were seen at 1000 *μ*m past the crush site (Fig 2A; S1 Fig), consistent with prior reports [48]. This contrasts with animals stimulated with ACB 1:4 in which six of nine rats demonstrated long-distance axon extension (Fig 2A, 2B and 2D; S3 Fig). Among these animals, 28-fold and 52-fold more RGC axon labeling was noted at 250 *μ*m and 1000 *μ*m past the crush site compared with unstimulated controls, respectively (p < 0.001, p < 0.05; two-way ANOVA with Tukey’s multiple comparisons test; S3 Table). Most axons decussated at the optic chiasm while few traveled ipsilaterally (Fig 2B and 2D), as occurs in developmentally normal rodents. Importantly, only rare RGC axon labeling was observed in animals stimulated with the SCB 1:1 waveform (S2 Fig), indicating that the driving force behind axon galvanotaxis is charge asymmetry, resulting in asymmetric duration of opposing directional EFs, rather than the simple presence of electrical stimulation. Similarly, only a few extended axons were noted to grow past the crush site in animals stimulated with ACB 4:1 (Fig 2A and 2C; S4 Fig), validating our prior in vitro findings that pulse width exerts a stronger influence on axon growth than pulse amplitude [46]. In fact, after 10 days of stimulation with ACB 4:1 (ie, the inverse of ACB 1:4; see Fig 2C), some axons appeared to turn back towards the globe. Altogether, our work indicates that electrical stimulation with ACB 1:4 directs long-distance RGC axon regeneration after optic nerve crush injury.

### Preserved RGC viability with biphasic electrical stimulation

**Fig 3:**
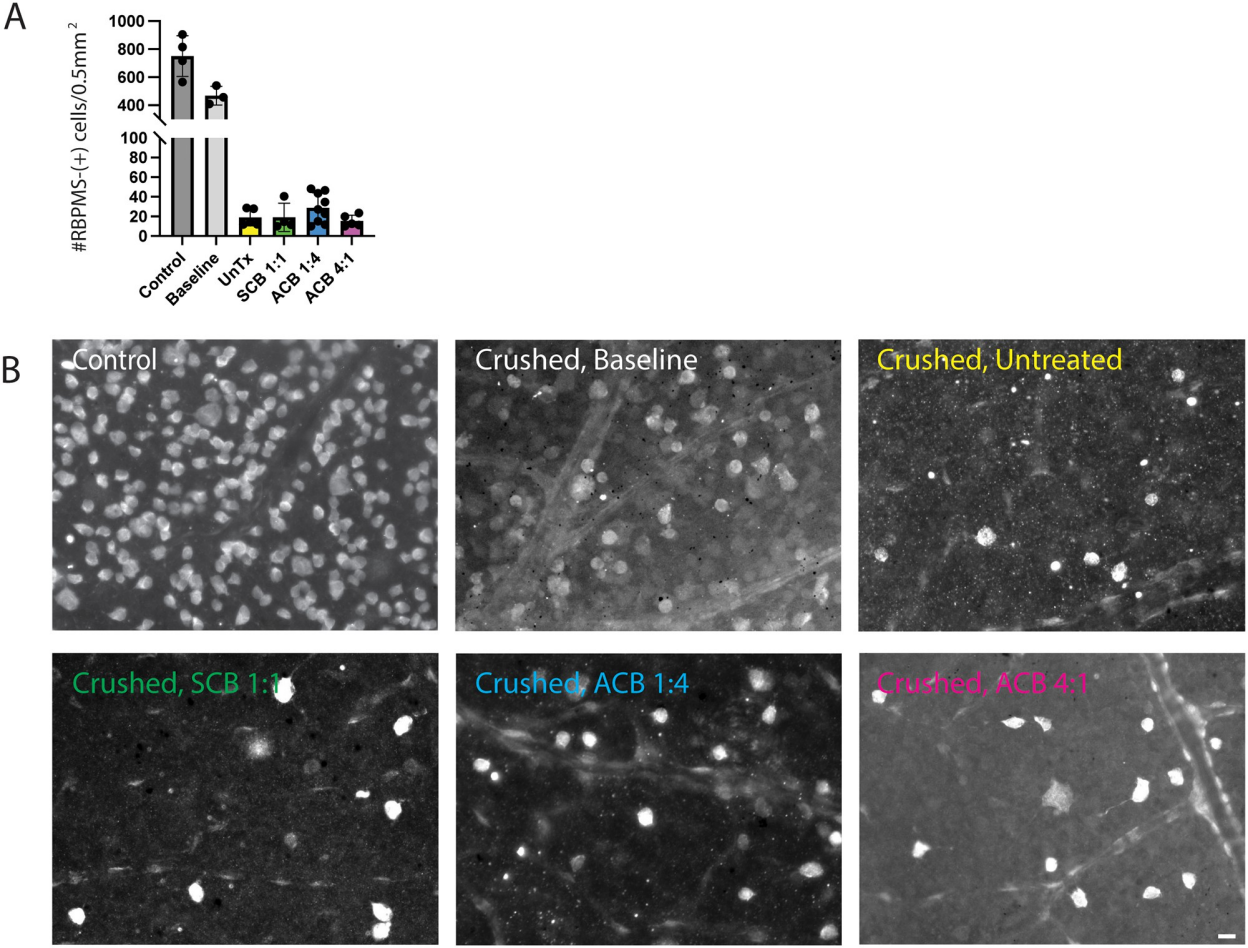
Preserved retinal ganglion cell (RGC) viability with biphasic electrical stimulation. Whole-mount retinas processed for RBPMS immunohistochemistry demonstrated no significant difference in RGC density in crushed, electric field-treated animals compared with crushed, untreated (UnTx) controls. Scatter dot plot of RBPMS cell density; One-way ANOVA with Tukey’s multiple comparisons test, error bars, STD. Scale bar, 20 *μ*m. ACB, asymmetric charge-balanced stimulation; SCB, symmetric charge-balanced stimulation.

At baseline, ie, 1 week after crush injury and electrode placement but before initiation of EF stimulation, whole-mount immunohistochemistry demonstrated a near 50% decrease in the number of RNA Binding Protein Multiple Splice (RBPMS)-positive RGCs per area compared with uncrushed controls (Fig 3). This number continued to decline over time. At 7 weeks post crush injury (6 weeks of stimulation), the average RGC density was 2-fold higher in EF stimulated animals compared to untreated controls (Fig 3, S4 Table). This increase, however, was not statistically significant. Similar to our prior in vitro findings [46], this indicates that electrical stimulation does not compromise RGC viability.

### Biphasic stimulation with ACB 1:4 mediates target-specific axon regeneration

**Fig 4:**
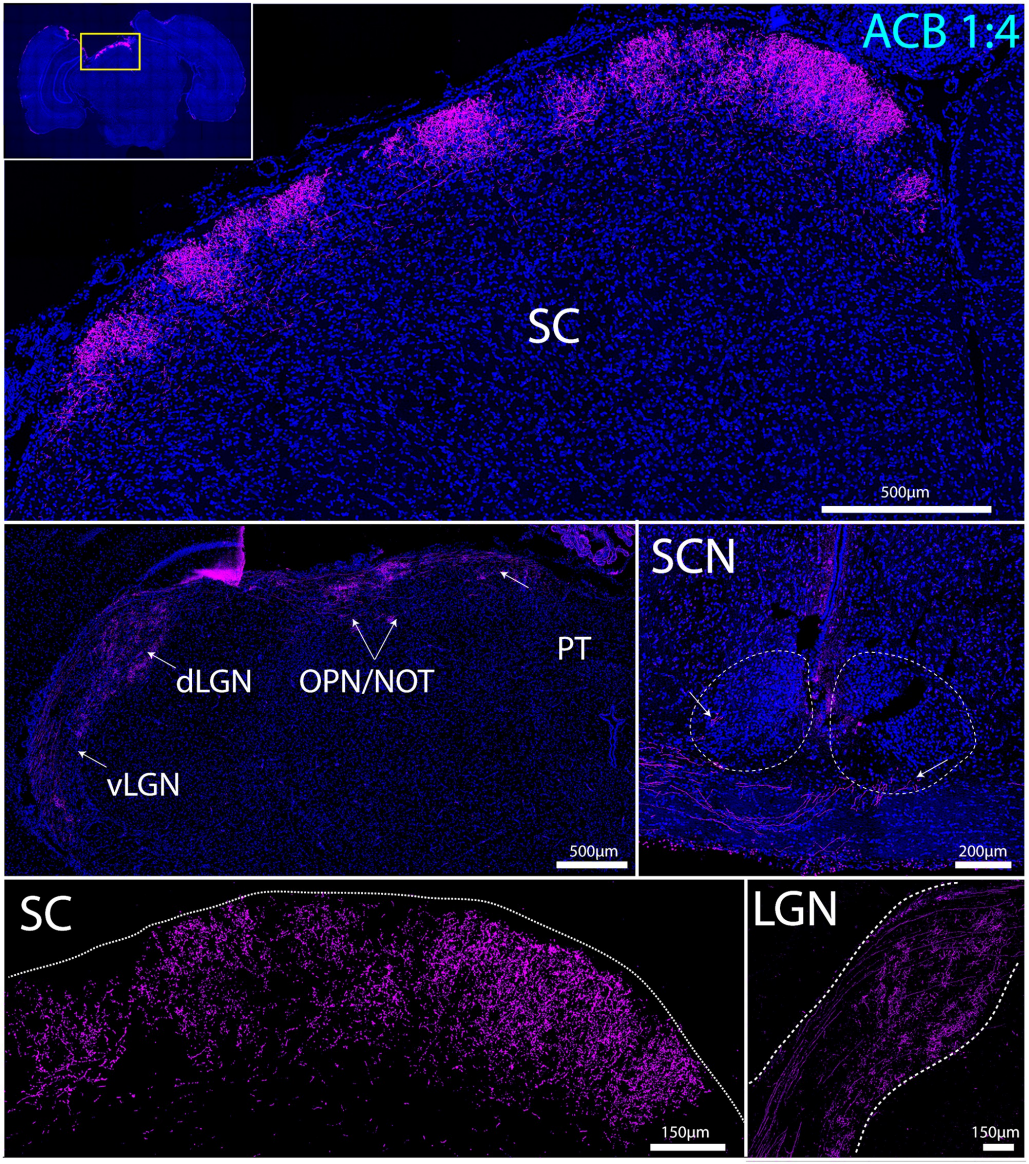
Biphasic stimulation with asymmetric charge-balanced (ACB) 1:4 stimulation mediates target specific regeneration. In ACB 1:4-treated animals, cholera toxin B (CTB)-labeled RGC axons (green) can be seen projecting to subcortical visual targets, including bilateral SCN and contralateral vLGN, IGL, dLGN, OPN, NOT, MPT, PPT, and SC. SCN, suprachiasmatic nucleus; vLGN, ventral lateral geniculate nucleus; IGL, intergeniculate leaflet; dLGN, dorsal lateral geniculate nucleus; OPN, olivary pretectal nucleus; NOT, nucleus of the optic tract; MPT, medial pretectal nucleus; PPT, posterior pretectal nucleus; SC, superior colliculus.

RGCs project to over 40 different regions in the brain to relay visual information. In the rodent brain, most RGCs project to either the superior colliculus (SC), which controls gaze, or to the lateral geniculate nucleus (LGN), which relays visual information to the primary visual cortex. RGC fibers also project to the suprachiasmatic nucleus (SCN), which controls circadian rhythm, the olivary pretectal (OPN) nucleus, which controls pupillary response, and the nucleus of the optic tract (NOT) which initiates or maintains horizontal gaze [49, 50]. Restoration of function after nerve injury requires interventions that can direct axons to grow to these subcortical targets. Given that animals stimulated with ACB 1:4 demonstrated long-distance axon growth past the optic chiasm, we investigated whether these RGC axons targeted appropriate structures in the diencephalon. After 6 weeks of stimulation of eight animals with ACB 1:4, cholera toxin B (CTB)-labeled RGC axons were observed projecting to bilateral SCN in one animal, the contralateral SCN and OPN in three animals, and the contralateral SC in five animals (Fig 4, S5 Table). In four of these animals, RGC axons also were observed in the contralateral dorsal and ventral lateral geniculate nucleus (dLGN and vLGN), the contralateral intergeniculate leaflet (IGL), and the contralateral NOT. Altogether, axons were observed projecting to at least one subcortical target in all but two of the eight animals. Of note, the axons appeared to laminate to the appropriate layer of the SC; there was no evidence of off-target regeneration to non-visual regions. In contrast, no RGC axons were observed to project to subcortical targets in untreated animals (S5 Fig).

### ACB 1:4 stimulation increases intracellular calcium concentrations in RGCs near cathode

**Fig 5:**
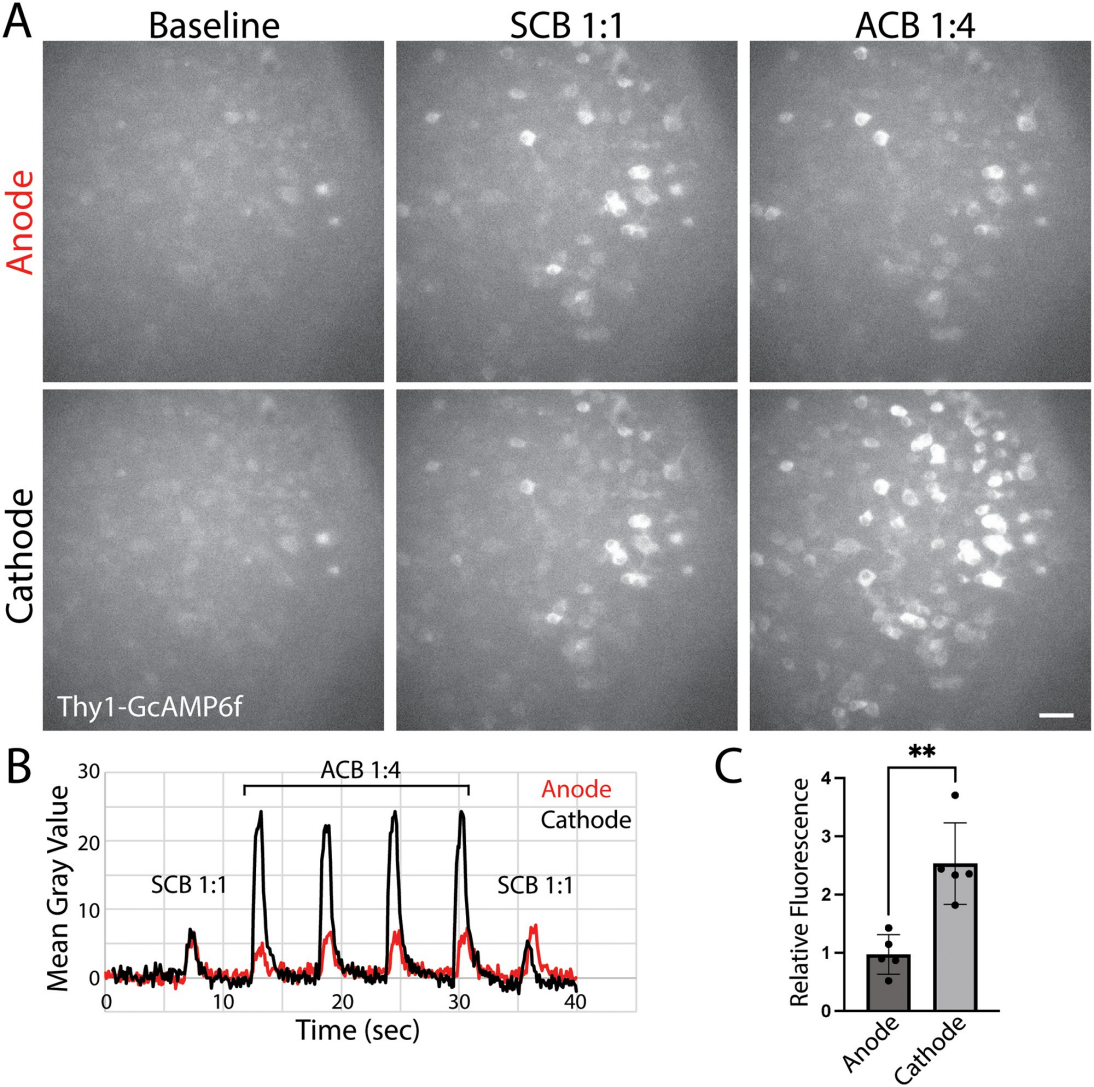
Asymmetric charge-balanced (ACB) 1:4 stimulation increases intracellular calcium concentrations in RGCs near cathode rather than anode. (A) Thy1-GcAMP6f wholemounts were exposed to SCB 1:1 followed by ACB 1:4 followed by SCB 1:1 waveforms. Then the polarity of the electrodes was inverted. Representative images of retina near the cathode versus anode shown. Scale bar 40 *μ*m. (B) Sample recordings of mean gray value at anode (red) versus cathode (black). (C) Average ratio of ACB 1:4 to SCB 1:1 mean grey value at anode versus cathode. N = 5 retinas, average 1:4 to 1:1 ratio at anode was 0.97 + 0.33, cathode 2.53 + 0.7; Student t-test, * p < 0.01, error bars, STD.

Prior reports suggest that EFs control cellular behavior by modulating local intracellular calcium concentrations [15]. Specifically, EF have been shown to activate more voltage-gated calcium channels (VGCCs) on neurite terminals that face the cathode than the anode [51–53]. This leads to increased activation of calcium-sensitive signaling pathways like RhoGTPase Rac1 (Ras-related C3 botulinum toxin substrate 1) and Cdc42 (cell division control protein 42) in cathodic-facing neurites. As Rac1 and Cdc42 promote actin polymerization, increased activity in cathodic-facing neurites leads to faster neurite extension towards the cathode than the anode. To test whether this could help explain how EFs directed target-specific RGC axon regeneration in rats, whole retinal explants from Thy1-GcAMP6f mice were exposed to SCB 1:1 or ACB 1:4 (Fig 5A) [44]. In RGCs near the anode, near equivalent responses were seen with ACB 1:4 stimulation compared with SCB 1:1 stimulation. In contrast, when the polarity of the EF was reversed, leading the same cells to now sit near the cathode, 2.5-fold more calcium influx was seen with ACB 1:4 stimulation compared with SCB 1:1 stimulation (Fig 5B and 5C; N = 5 retinas, average 1:4 to 1:1 ratio at anode was 0.97 + 0.33, cathode 2.53 + 0.7; Student t-test, p < 0.01). These experiments demonstrate that tissues closer to the cathode have increased calcium influx with ACB 1:4 stimulation compared to cells that sit near the anode. Given that the cathode is implanted in the optic tract of our rats, these experiments suggest that one mechanism by which in vivo EF stimulation with ACB 1:4 could direct axon regeneration is by inducing local calcium influx into RGC axonal terminals, driving neurite extension towards the intracranial cathode.

### RGCs are more responsive to SCB 1:1 and ACB 1:4 than ACB 4:1 stimulation

**Fig 6:**
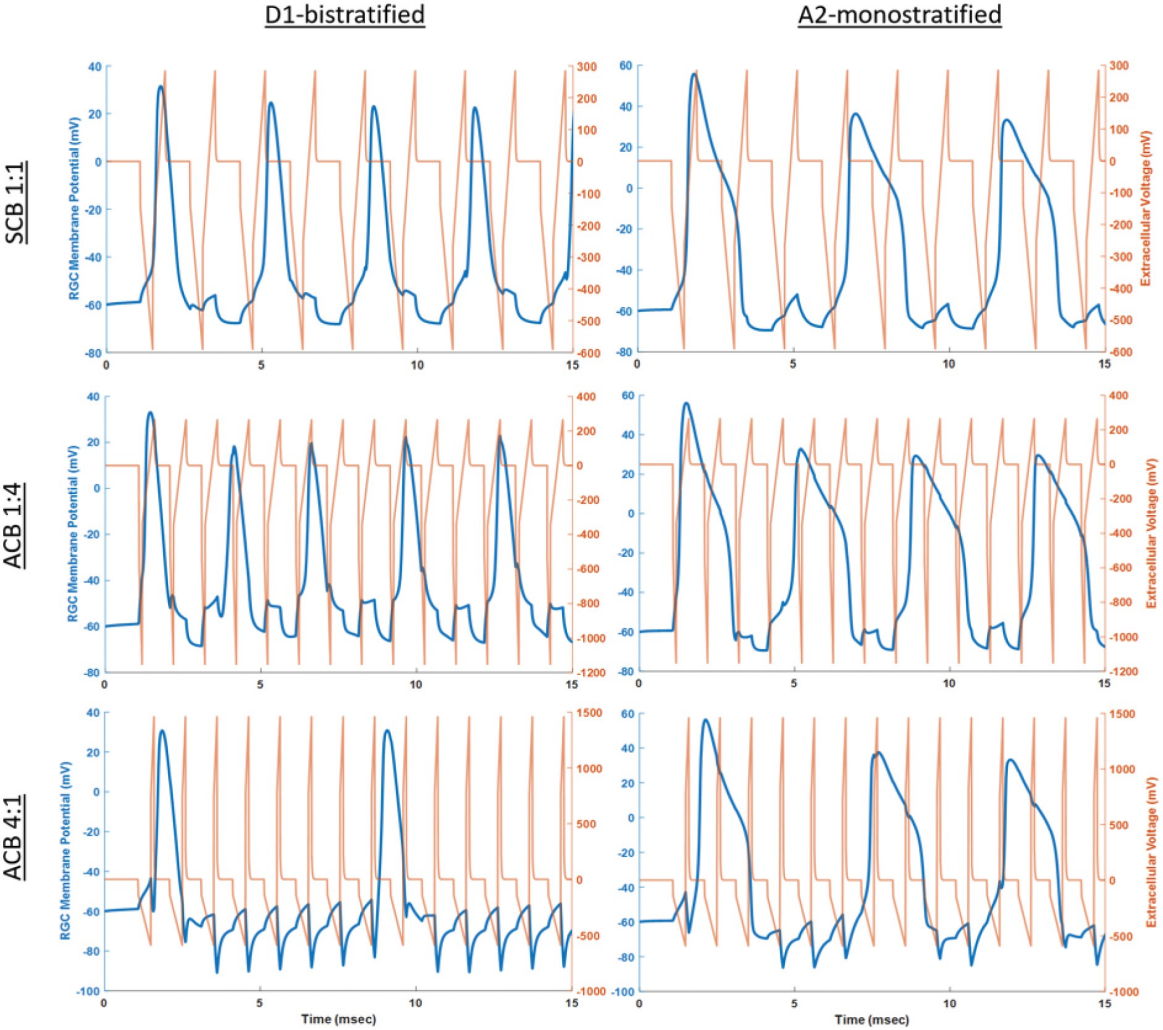
Biphasic stimulation elicits action potentials in retinal ganglion cells (RGCs). Time course of the membrane potential response for two RGC subtypes when stimulated with all three waveforms. The extracellular potential generated near the soma is computed using AM and the cell membrane response is computed in the NEURON simulation environment. Although the D1 cells can better sustain repetitive firing at high frequencies, both RGC subtypes can fire very fast at these amplitudes, across all waveforms. However, the simulations suggest a preferential activation in the D1 subtype when using SCB 1:1 and ACB 1:4 over ACB 4:1, with over 120% increase in the sustained firing rate. ACB, asymmetric charge-balanced waveforms; SCB, symmetric charge-balanced waveforms.

Previous investigators have shown that neural activity enhances RGC axon regeneration after crush injury [48]. We postulated that an alternative mechanism through which EFs could direct RGC axon regeneration is by activating RGCs. To test this hypothesis, we employed previously developed RGC models to simulate RGC responses to ACB stimulation [31]. Because different RGC subtypes are sensitive to different stimulation frequencies, we modeled two types of RGCs that represent different ends of this spectrum: the D1 bistratified subtype, which has been shown to be responsive to high-frequency electrical stimulation and the A2 monostratified subtype, which is responsive to lower frequency electrical stimulation [32, 33]. AM/NEURON (see Methods) computational modeling indicates that, with continuous SCB 1:1, ACB 1:4 and ACB 4:1 stimulation at the same frequencies that were employed in vivo, the cathodic pulse elicits an action potential every 3.25, 3.0, and 6.8 msec in D1 and 4.85, 3.95, and 6.35 msec in A2 RGCs, respectively (Fig 6). These results are similar to prior reports [54] and suggest that there may be preferential activation for different phase sequences, as seen in the D1 subtype where over a 120% faster firing rate was observed with either SCB 1:1 and ACB 1:4 stimulation compared with ACB 4:1 stimulation [30]. Thus, inefficient RGC activation could explain why no regeneration was seen with ACB 4:1 stimulation. However, given that only ACB 1:4, and not SCB 1:1, stimulation conferred an appreciable regenerative response after crush injury in adult rats, our results also suggest that RGC activation is not the only mechanism through which ACB 1:4 mediates axon regeneration. Rather, the different regenerative responses seen between these different waveforms is more likely a consequence of EF gradients inducing local calcium influx (Fig 5) into neurites [52, 53], driving axon extension towards the intracranial electrode.

### Biphasic stimulation with ACB 1:4 directs partial recovery of RGC electrophysiology

**Fig 7:**
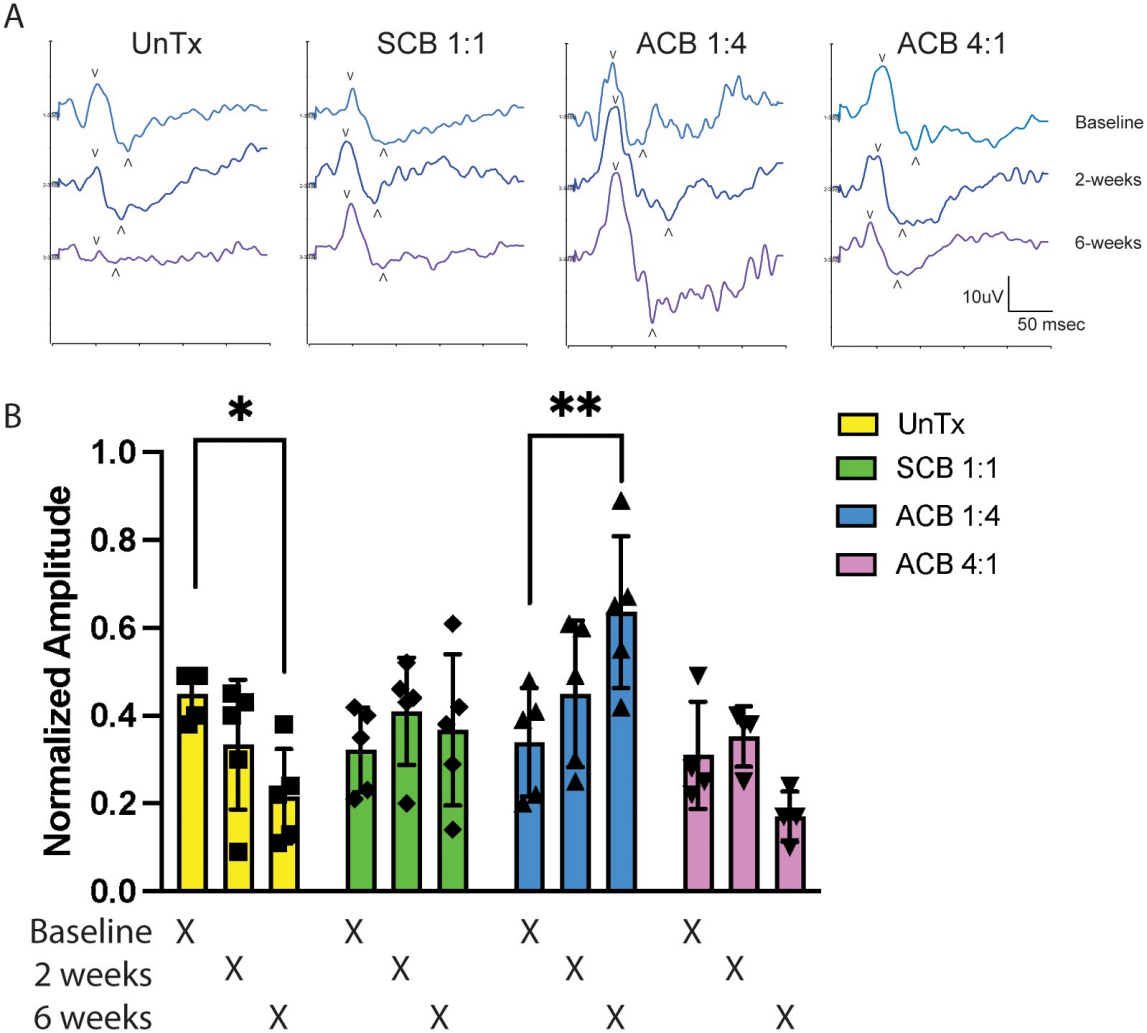
Biphasic stimulation with asymmetric charge-balanced (ACB) 1:4 waveforms mediates partial recovery of retinal ganglion cell (RGC) function. Serial pattern electroretinogram (PERG) recordings were performed in animals after optic nerve crush injury. (A) Representative PERG recordings with a 15-point smoothing filter applied. Baseline: one week after electrode placement and crush injury but before initiation of electrical stimulation. ∨, P50 peak; ∧, N95 trough. (B) Normalized N95 amplitude (left eye: right eye) over time in each stimulation group; two-way ANOVA with Tukey’s multiple comparisons test; * p < 0.05; ** p < 0.01, error bars, STD). SCB, symmetric charge-balanced.

To assess whether treatment with electrical stimulation after optic nerve crush injury restores RGC function, serial pattern electroretinogram (PERG) recordings were performed at baseline (1 week after crush injury, before initiation of treatment), after 2 weeks of electrical stimulation, and after 6 weeks of electrical stimulation in the same animal (Fig 7). To account for variability in signal response from anesthesia, recordings in the left eye were normalized to the right eye. At baseline, the normalized N95 amplitude decreased to an average ratio of 0.45 (+/- 0.02 SEM) in the untreated group. No difference in baseline measurements was detected between untreated animals and animals assigned to any of the treatment groups (S6 Table). Two-way ANOVA detected significant time by group interaction (p < 0.0001). Post-hoc analysis using Tukey’s multiple comparisons test demonstrated a significant decline in PERG amplitudes in the untreated group over time. In contrast, animals treated with ACB 1:4 demonstrated a significant 1.8-fold increase in their normalized PERG amplitude after 6 weeks of stimulation compared with baseline (baseline 0.37 +/- 0.04 vs 6-weeks 0.68 +/- 0.07, SEM, p < 0.05). This indicates that ACB 1:4 stimulation conferred partial restoration of RGC function. Although SCB 1:1 stimulation failed to significantly increase PERG amplitudes over time (baseline 0.32 +/- 0.05 vs 6- weeks 0.52 +/- 0.11 SEM), these animals did not demonstrate the continued loss of function seen in untreated animals and ACB 4:1- stimulated animals, suggesting a weak neuro-protective effect.

**Fig 8:**
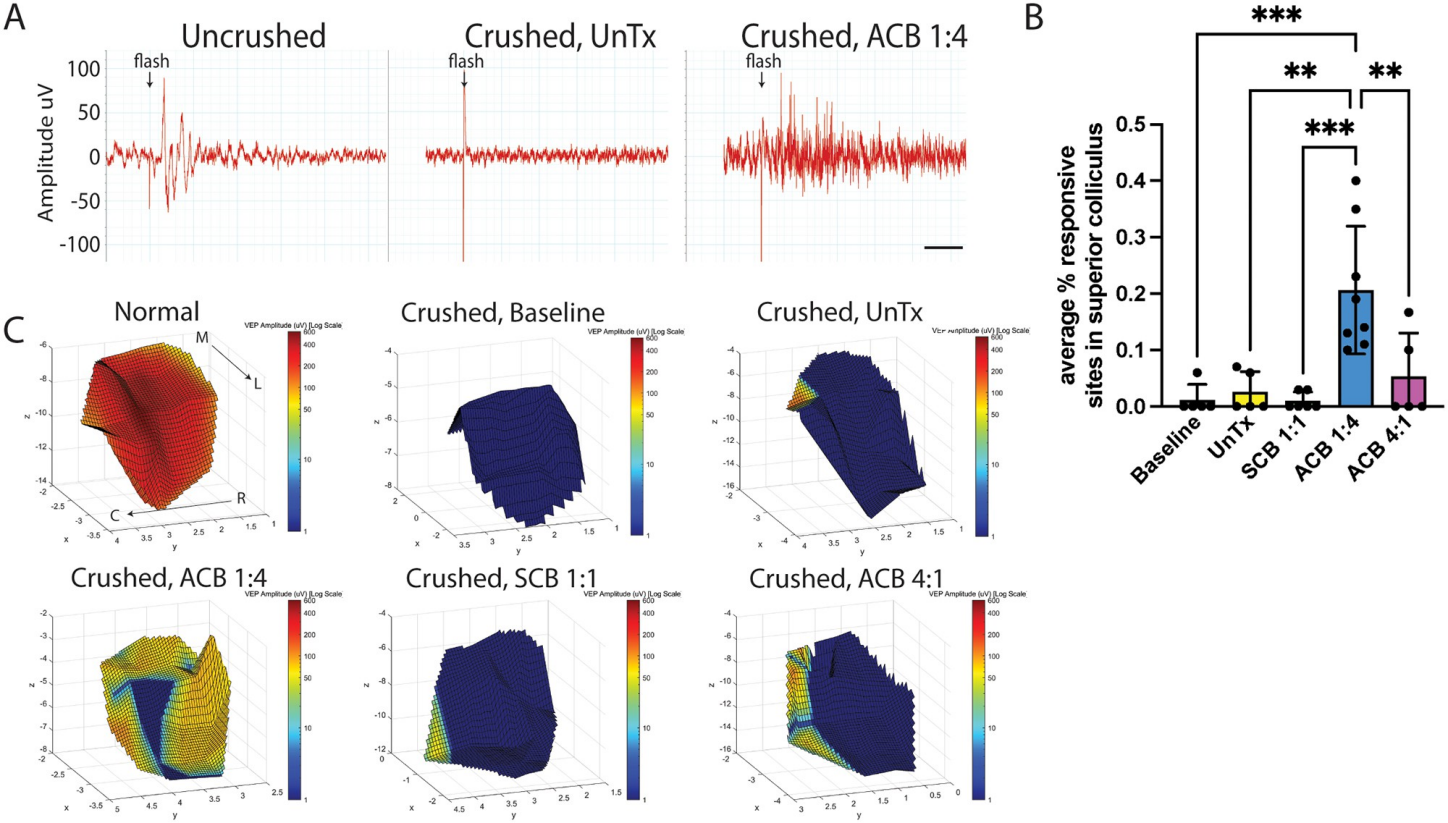
Biphasic stimulation with asymmetric charge-balanced (ACB) 1:4 waveforms mediates partial recovery of light-evoked local field potential recordings in the superior colliculus. Stereotactic-guided local field potential (LFP) responses to full-field light stimulation were measured in the contralateral superior colliculus (SC). (A) Sample recordings. (B) Average percent of sites demonstrating a response. Baseline: one week after electrode placement and crush injury but before initiation of electrical stimulation. Error bars, STD. One-way ANOVA with Tukey’s multiple comparisons test; ** p < 0.01, *** p < 0.001. (C) 3D rendering of the distribution of LFP responses in the SC of representative animals. M, medial; L, lateral; R, rostral; C, caudal. SCB, symmetric charge-balanced.

To demonstrate that visual information is being transmitted by RGCs to target structures in the brain, we performed stereotactic-guided, light-evoked local field potential (LFP) recordings in the contralateral superior colliculus (SC) [36, 55]. On average, 2.67% +/- 1.64% (SEM) of areas in the SC demonstrated a response to full-field light stimulation in untreated animals (Fig 8; S7 Table). These rare responses (2 sites) were detected in two of the five untreated animals. Remarkably, all eight animals that underwent 6 weeks of stimulation with ACB 1:4 demonstrated a positive response to full-field light stimulation (Fig 8, S7 Table). On average, 20.69% +/- 4% of sites (SEM) in ACB 1:4-treated animals demonstrated a positive response, ranging from 10% to 40% sites out of an average of 31 sites tested per animal. However, the average amplitude of 81.78 +/- 18.1 *μ*V and the average latency of 113 +/- 31.4 msec in ACB 1:4 treated rats were lower and longer than in normal age-matched controls (amplitude 223.7 +/- 93.7 *μ*V; latency 31.4 +/- 3.3 msec; N = 3; mean +/- SD; S7 Table), respectively. Similar to untreated animals, rare responses were recorded in SCB 1:1 and ACB 4:1 groups (1% +/- 1% and 5% +/- 3% of sites, respectively). Of note, no response was detected in baseline animals (evaluated one week after crush but before initiation of electrical stimulation). Taken together, our PERG and LFP recordings suggest that ACB 1:4 directs visual restoration rather than visual preservation.

### Biphasic stimulation with ACB 1:4 failed to mediate recovery of pupillary function

Direct pupillary light reflex (PLR) testing was performed to measure recovery of pupillary function. No significant differences in pupillary constriction were observed among the different treatment groups after 10 seconds of photic stimulation (S6 Fig). Although some RGC axons were found to successfully target the OPN in ACB 1:4-treated rats (Fig 4), lack of recovery of the PLR response indicates that ACB 1:4 mediated regeneration was insufficient to confer functional recovery.

### Biphasic stimulation with ACB waveforms failed to mediate recovery of visual behaviors

To assess whether ACB 1:4 stimulation conferred recovery of visual behaviors, we challenged rats with the visual cliff test and optokinetic reflex (OKR) testing (S8 Table). Two of eight rats dismounted onto the correct side in the ACB 1:4 stimulation group. This did not differ significantly from untreated animals or animals treated with SCB 1:1 or ACB 4:1 (average shallow dismounts of 40%, 33.3% and 50%, respectively). Similarly, none of our rats, stimulated or untreated, demonstrated head tracking with OKR testing (UnTx, N = 5; SCB 1:1, N = 6; ACB 1:4, N = 8; ACB 4:1, N = 4). Altogether, electrical stimulation failed to mediate recovery of behavioral visual function.

### Functional recovery with biphasic stimulation represents RGC axon regeneration rather than spared axons

Several findings suggest that RGC axon labeling past the crush site and functional visual recovery are a result of RGC axon regeneration as opposed to spared axons. Firstly, CTB-647 labeling of baseline animals (1 week after crush injury but before initiation of electrical stimulation) demonstrated no labeling of RGC axons past the crush site (S7 Fig), consistent with previous reports [48]. Second, serial PERG recordings demonstrated improvement over time within the same animal (Fig 7), further suggesting a case of restoration of visual function. Finally, LFP recordings performed in animals at baseline demonstrate no response in the SC (Fig 8), indicating that the responses recorded after 6 weeks of ACB 1:4 stimulation likely represent a recovery of function.

### Strength and distribution of the electric field along the optic nerve is dependent on electrode position

We observed variability in how effective the ACB 1:4 waveform was at directing RGC axon regeneration (S3 Fig). A possible explanation for this variability is that the voltage gradient generated along the optic nerve is sensitive to electrode position. To interrogate this, we performed 3D computational AM/NEURON modeling of our stimulation system.

**Fig 9:**
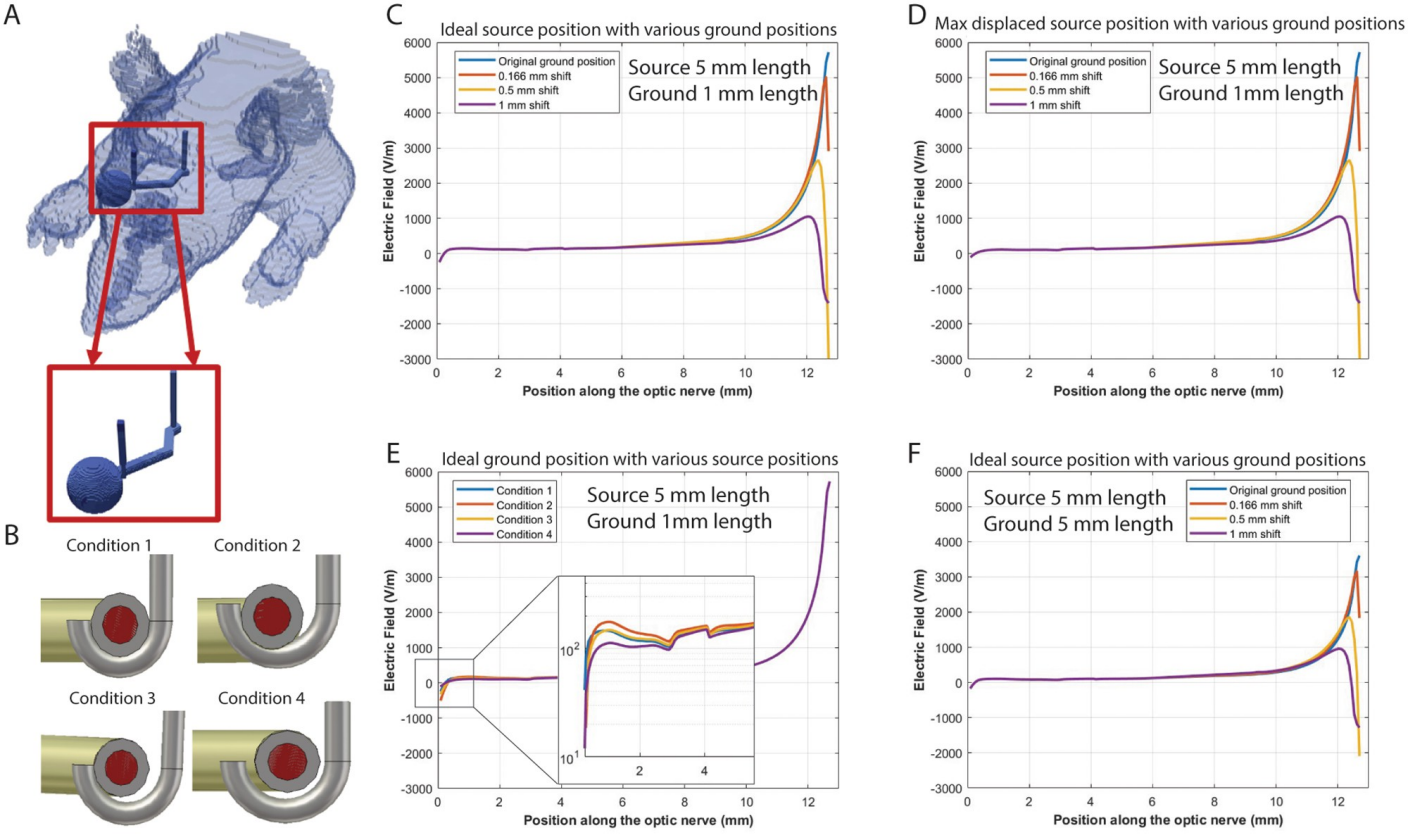
Electric gradient along the optic nerve is sensitive to ground electrode positioning. (A) AM admittance method was used to generate a 3D computational model of the electric gradient along the optic nerve. Inset shows “J” shaped source electrode wrapped around the optic nerve while the ground electrode pierces the contralateral optic tract. (B) Different source electrode positions considered. Electric gradient along the optic nerve and tract with ideal source position and various displacement in ground position (C; Condition 1); with maximum displacement in source position and various displacement in ground position D; Condition 4); with ideal ground position and various displacement in source position (E; Conditions 1–4); with ideal source position and various displacement in ground position (F; Condition 1) but with larger ground electrode than in (C).

A 3D model of the rat head and orbit was made using the AM/NEURON, a numerical electromagnetic method that partitions tissue into an admittance network and computes the EFs generated from a current-controlled stimulation pulse [22]. To investigate the effect of electrode misplacement on the voltage gradient generated along the optic nerve, we modeled the source and the ground electrode in various positions (Fig 9). Specifically, the source electrode was modeled as being in direct contact with the optic nerve for half its circumference (1.3 mm of direct contact), a quarter of its circumference (0.65 mm of direct contact), at a single point (0.21 mm of direct contact), and 0.5 mm away without any direct contact (Fig 9B). The ground electrode was modeled as piercing the optic tract or shifted 2 voxels (166 *μ*m), 6 voxels (0.5 mm), or 12 voxels (1 mm) laterally. A total of 16 different permutations were tested.

We calculated the magnitude of the EF generated along the optic nerve and tract toward the brain from *E* = *ΔV*/*Δd*, in which *ΔV* is the voltage difference between two adjacent points and *Δd* is the distance between them (83 *μ*m). In the ideal case, where the source electrode makes maximum contact with the optic nerve and the ground electrode pierces the optic tract, the EF has a relatively flat profile starting from the stimulation electrode until, within 2 mm of the ground electrode, it increases drastically (Fig 9C). This change occurs just beyond the optic chiasm and likely results from several factors including the sudden change in surrounding resistivity due to the lack of CSF in the distal optic tract and small electrode size. Incrementally shifting the ground electrode away from its ideal position while maintaining ideal contact with the optic nerve leads to significant decreases in the maximum amplitude of the EF: a 1 mm shift was associated with an 82% decrease in EF amplitude. Conversely, when the ground electrode was in the ideal position, but the source electrode made less contact with the optic nerve, 30–50% change in EF was observed (Fig 9D and 9E). Overall, our data suggest that variability in ground and source electrode positioning could account for some of the variability observed in our animal experiments.

One possible explanation for why the EF is sensitive to ground positioning is that the surface area of the ground is 5-fold smaller than that of the source electrode. In our setup, the surface area of the source electrode was intentionally designed to be large (5 mm of scraped insulation) to ensure contact with a mobile optic nerve. Conversely, the small ground surface area (1 mm scraped insulation) was chosen to minimize off-target effects in the densely packed CNS. Indeed, when the ground electrode was modeled to have the same surface area as the source electrode, the sensitivity of the EF to the ground electrode position decreased (Fig 9E and S8 Fig); instead of an 88% change, the EF decreased by 55% when the ground was shifted by 1 mm, similar to variations observed with shifts in the source electrode position. Although increasing ground size may be an attractive strategy for building a more robust stimulation system, larger ground electrodes cause smaller electric fields (S8 Fig) that may diminish EF effects on axon growth.

## Discussion

To date, most approaches for promoting axon regeneration exploit genetic tools to selectively upregulate dormant signaling pathways that were active during development, with the aim of reverting neurons back to a developmental or growth state. A potential limitation associated with these approaches is that they do not provide growing axons with directional cues, which may explain why examples of stalled growth at the optic chiasm or aberrant regeneration have been reported in the literature. For highly organized structures like the CNS, restoration of function requires approaches that not only drive axon growth but also “steer” axons towards intended targets. Here, we demonstrate that applying an external guidance cue in the form of an electric field was successful at directing target-specific axon regeneration (Fig 4) and conferring partial recovery of visual function (Figs 7 and 8). Notably, our approach delivered these gains without the need for concurrent genetic modulation or signs of aberrant growth.

The concept that external cues can override cell intrinsic barriers to axon regeneration is well documented. For example, transected RGC axons fail to grow into CNS nerve grafts but readily sprout axons into peripheral nerve grafts [56, 57]. These experiments not only indicate that providing a permissive environment for regeneration could be a viable approach for directing axon growth but also that environmental cues can override cell intrinsic barriers to axon regeneration. However, recapitulating the symphony of extracellular molecular gradients that directed tissue patterning and axon growth during development within an adult animal has been exceedingly difficult. One reason for this failure could be that axons need these extracellular guidance molecules to be expressed in not only spatial but also temporal gradients [11, 13, 58, 59]. EF stimulation offers a unique solution to this dilemma. Not only can exogenous EFs modulate the direction of axon growth, as has been demonstrated with many different neuron subtypes in tissue culture, but the strength, location, and duration of EF stimulation can be readily manipulated in vivo by controlling the location of electrode placement, modulating the frequency, amplitude, and pulse width of the stimulation waveform, and controlling the duration of stimulation. In other words, EF stimulation can be easily titrated to need.

### Axon growth from induced voltage gradients

The mechanism through which external voltage gradients (EFs) elicit specific cellular behaviors like axon growth is likely to be multi-factorial. Prior work by Lim et al. demonstrated long-distance RGC axon regeneration after optic nerve crush injury in adult mice that underwent treatment with biased visual activity [48]. Given that electrical stimulation readily elicits action potentials in neurons, RGC activation is one mechanism through which ACB 1:4 stimulation is likely to have directed RGC axon growth in our experiments. The finding that ACB 1:4 is more efficient at activating RGCs than ACB 4:1 (Fig 6), and that ACB 1:4 stimulation was associated with RGC axon regeneration while ACB 4:1 was not (Fig 2), lends further support to this hypothesis.

Several findings, however, suggest that neural activation alone is insufficient to explain our results. Specifically, EF-directed RGC axon regeneration in our study did not require concurrent mTOR activation or visual deprivation of the contralateral eye, co-treatments without which the positive outcomes reported by Lim et al. were limited. The difference between their findings and ours could stem from differential levels of cellular activation, with applied EFs being more efficient. Additionally, our modeling and work by others [54] showed that SCB 1:1 and ACB 1:4 waveforms were equally effective at eliciting an action potential in RGCs (Fig 6). If the regenerative response seen in ACB 1:4 was solely based on RGC activation, a similar amount of regeneration should have been observed with SCB 1:1 as ACB 1:4 stimulation.

Other proposed mechanisms for EF directed electrotaxis include focal activation of voltage-gated calcium channels in neuronal foot processes [52], leading to local activation of calcium-dependent signaling pathways (e.g. Rac1/Cdc42) [18, 51], that then preferentially drive actin polymerization in cathode oriented neurites. In support of this, we showed that cells near the cathode demonstrated 2.5-fold larger influx of calcium than cells that sat near the anode with ACB 1:4 stimulation (Fig 5). This differential effect was 1) not seen with SCB 1:1 stimulation and 2) was observed when electrodes were placed 8.5 mm apart. In rats, stimulation electrodes are approximately 10 mm apart: the anode sits in the orbit behind the globe while the cathode sits near contralateral optic tract. In other words, the cathode is at least 10 mm away from the axon hillock or RGC action potential initiation center. Thus, an alternative, although not exclusive, explanation is that ACB 1:4 stimulation could be inducing focal calcium influx into RGC axon terminals, thereby not just “driving” but also “steering” axon growth. Calcium influx could be mediated by L-type voltage gated calcium channels, which are readily found in RGC axons [53]. Local calcium influxes could explain why regenerated nerves in ACB 1:4 treated animals were not stalled at the optic chiasm and why no evidence of aberrant regeneration was seen.

In addition to cellular activation, pulsed EFs have been proposed to control cellular migration and alignment by altering the extracellular matrix [60]. For example, EFs have been shown to alter calcium distribution within Matrigel sheets [61]. When Schwann cells were seeded onto Matrigel pretreated with EFs one week earlier, cells aligned perpendicularly. Collagen fibers have also been shown to align with EF stimulation [62]. Altering the extracellular milieu thereby creating a permissive environment for RGC axon extension is another mechanism through which ACB 1:4 stimulation could direct axon growth after crush injury.

### Not all waveforms are created equal

Interest in developing EF stimulation into a technology to modulate cellular behavior and direct tissue regeneration was first introduced in the 1920s [63, 64]. The premise for this approach stemmed from a large body of literature showing that electric potentials are generated naturally by the body and play a pivotal role in directing tissue patterning during development [65] and wound healing after injury [15]. So why have these approaches largely failed to translate into clinical treatments? One major reason is that most prior approaches employed direct current (DC) stimulation. Although effective at directing CNS axon regeneration in the spinal cord of lampreys and guinea pigs [66–68], DC stimulation can be applied only at low amplitudes or for short intervals before charge accumulation causes tissue damage [20].

To circumvent some of the limitations associated with DC stimulation, we employed a unique subclass of biphasic waveforms—the asymmetric charge-balanced waveform—to guide RGC axon growth. ACB waveforms are charge balanced and, thus, safer than DC but retain the ability to generate the field needed to direct axon growth through asymmetry between their anodic and cathodic phases. It is exactly this asymmetry that enabled ACB 1:4 stimulation to be more effective at guiding axon growth than SCB 1:1. The tradeoff between DC and ACB stimulation, however, is efficacy as the pulse width of ACB waveforms, which has significant influence on axon growth [46], will always be shorter than that of DC.

In addition to efficacy and safety, waveform morphology is a critical consideration when designing EF-based therapies. While SCB 1:1 was ineffective at driving axon growth, stimulation with symmetric waveforms has been shown to be protective, promoting survival of RGCs after optic nerve transection [16]. Differential cellular responses evoked by different waveforms on different cell populations has been previously described [15] and provides an opportunity to tailor EF treatment to tissue need.

### EF stimulation confers partial recovery of electrophysiologic function

Although electrical stimulation was able to confer partial recovery of electrophysiologic function, it failed to restore normal visual behaviors (OKR and visual cliff) and pupillary response. Several explanations may account for this finding. First, although EF stimulation with ACB waveforms did not compromise RGC viability (Fig 3), in agreement with our prior tissue culture work [46], it did not confer statistically significant increase in RGC survival either. This could be a result of the frequencies we tested (650–1000Hz), which are higher than those previously shown to promote RGC survival [16], or a result of late treatment initiation (5–7 days after crush injury). As a result, ACB 1:4 stimulation was only able to drive axon growth of RGCs that survived crush injury—approximately 13% of the RGC population—a number insufficient to restore behavioral and pupillary function. In fact, this likely is another reason for the failure of DC stimulation to translate into widespread clinical use. Unlike ACB 1:4, DC stimulation decreases RGC viability [46]. Negative effects of DC stimulation on RGC survival would have confounded gains conferred DC stimulation to promote axon growth. Nevertheless, given the breakthrough discovery of neuro-protective molecules like CNTF and BDNF [69], it would be worthwhile to investigate if combining ACB 1:4 stimulation with these neuroprotective molecules could offer synergistic gains in function.

Another possible explanation for incomplete recovery of function with ACB 1:4 stimulation is that regenerated axons may be unmyelinated or form aberrant synapses with target neurons. LFP recordings in ACB 1:4-treated rats had decreased amplitude and longer latency compared with age-matched normal animals. As LFP recordings are a summation of local signaling, it is unknown if this finding represents signaling from a few normal-functioning RGC axons or many aberrantly signaling RGC axons. Bei et al. found regenerated axons in the optic tract to be unmyelinated, and, thus, we favor the latter possibility [4]. Ongoing investigations in our lab are aimed at characterizing the extent to which regenerated RGC axons are myelinated as well as the composition of the synaptic complexes in regenerated RGC growth cones.

Another possible explanation for our failure to restore visual behavior could be due to failure of specific RGC subtype regeneration. Performance on OKR relies on direction-sensitive RGCs in the accessory optic system [70–72] whereas the visual cliff test relies on binocular vision and involves both the dLGN and the primary visual cortex [73]. Deletion of Rbfox1, a family of ribonucleic acid binding proteins, led to loss of performance on visual cliff testing in aged mice [74]. Prior reports have shown that *α*RGCs represent the majority of RGCs that regenerate with PTEN deletion, possibly because of higher intrinsic mTOR activity [75, 76]. Although RGC axons could be observed to project to the dLGN in ACB 1:4-treated animals, perhaps levels were subthreshold compared with what is needed for functional recovery. It will be interesting to see if ACB 1:4 directs regeneration of a similar subset of RGC subtypes compared with strategies that modulate cell-intrinsic barriers to regeneration.

Finally, failure to restore visual behavior could result from failure of regenerated RGC axons to maintain retinal topography. Although our LFP recordings indicate that RGCs are transmitting information to the SC, and histology in our animals shows RGC axons projecting to both the SC and the LGN, it is unknown whether or not these cells are transmitting formed images. Computational modeling of the distribution of the EF induced by our electrode configuration shows a constant field along the optic nerve that increases at the level of the optic tract (Fig 9). Although this distribution is likely to help guide regenerating RGC axons to the diencephalon, we do not know if this gradient has the resolution to provide cues for proper lamination. However, lamination-specific regeneration has been reported by others and is thought to stem from developmental guidance cues still being present in the adult CNS [77]. Current efforts in our lab are directed at assessing whether the retinotopic map is maintained in ACB 1:4-treated animals.

In conclusion, breakthrough discoveries of molecules that mediate neuro-protection and activate signaling pathways that are capable of driving axon growth now generate a need for approaches to guide these regenerating axons to intended targets. Our work suggests that electrical stimulation could play an important role in facilitating target-specific regeneration of CNS neurons. Ultimately, translation of these findings into the clinical arena will require significant effort to demonstrate safety of long-term stimulation with EFs [78, 79]. These efforts are ongoing in our lab.

## Supporting information

S1 Fig Optic nerves of animals that received no treatment for 6 weeks (sacrificed 7 weeks post crush injury). Orthogonal images (20x magnification) of cholera toxin B-labeled optic nerves of untreated animals. Few axons seen past the crush site (asterisk). Scale bar, 250 *μ*m. UnTx, untreated.
(TIF)

S2 Fig Optic nerves of animals treated with symmetric charge-balanced (SCB) 1:1 waveforms for 6 weeks. (A) Orthogonal images (20x magnification) of CTB-labeled optic nerves of animals in the SCB 1:1 group. Few axons seen past the crush site (asterisk). Scale bar, 250 *μ*m. (B) Schematic of SCB 1:1 waveform.
(TIF)

S3 Fig Optic nerves of animals treated with asymmetric charge-balanced (ACB) 1:4 waveforms for 6 weeks. (A-I) Orthogonal images (20x magnification) of cholera toxin B-labeled optic nerves of animals in the ACB 1:4 waveform group. Many axons seen past the crush site (asterisk). (A1-I3) Select Z-stack images were collapsed and magnified from corresponding insets in (A-H) to reduce background noise and show the course of retinal ganglion cell (RGC) axons more clearly. Scale bars, 250 *μ*m. (J) Schematic of ACB 1:4 waveform. (K) Quantification of RGC axon density at 250 *μ*m intervals from the crush site after stimulation with various waveforms for 6 weeks (UnTx, N = 5; SCB 1:1, N = 4; ACB 1:4, N = 6; ACB 4:1, N = 4; error bars, SEM; * p < 0.05, ** p < 0.01, *** p < 0.001; two-way ANOVA with Tukey’s multiple comparisons test).
(DOCX)

S4 Fig Optic nerves of animals treated with asymmetric charge-balanced (ACB) 4:1 waveforms for 6 weeks. (A) Orthogonal images (20x magnification) of CTB-labeled optic nerves of animals in the ACB 4:1 group. Few axons seen past the crush site (asterisk). Scale bar, 250 *μ*m. (B) Schematic of ACB 4:1 waveform.
(TIF)

S5 Fig No axonal projections seen in sham animals. (A) In sham animals, no cholera toxin B (CTB)-labeled RGC axons can be seen projecting to subcortical visual targets, including bilateral SCN and contralateral vLGN, IGL, dLGN, OPN, NOT, MPT, PPT, and SC. SCN, suprachiasmatic nucleus; vLGN, ventral lateral geniculate nucleus; IGL, intergeniculate leaflet; dLGN, dorsal lateral geniculate nucleus; OPN, olivary pretectal nucleus; NOT, nucleus of the optic tract; MPT, medial pretectal nucleus; PPT, posterior pretectal nucleus; SC, superior colliculus.
(TIF)

S6 Fig Treatment with asymmetric charge-balanced (ACB) 1:4 waveforms for 6 weeks failed to mediate recovery of pupillary function. (A) Pupil circumference of an untreated animal (yellow circle) ACB 1:4 treated animal (blue circle) demonstrate no changed after 10 seconds of photic stimulation. (B) Quantification of pupillary change after 10 seconds of photic stimulation (UnTx, N = 7; SCB 1:1, N = 4; ACB 1:4, N = 7; ACB 4:1, N = 6; error bars, SEM; two-way ANOVA with Tukey’s multiple comparisons test). SCB, symmetric charge-balanced; UnTx, untreated.
(TIFF)

S7 Fig Optic nerves of baseline animals sacrificed 1 week after crush injury and before any stimulation with electric fields. Orthogonal images (20x magnification) of cholera toxin B-labeled optic nerves of baseline animals. Few axons observed past the crush site (asterisk). Scale bar, 250 *μ*m.
(TIF)

S8 Fig Increases in ground electrode size are associated with decreases in electric field. AM admittance method was used to generate a 3D computational model of the electric gradient along the optic nerve. Electric gradient along the optic nerve and tract with ideal source position (Condition 1, from Fig 7) and ideal ground position but with different size ground electrodes.
(TIF)

S1 Table Target stimulation parameters. Target stimulation parameters for each waveform.
(DOCX)

S2 Table Resistivity of different materials used in the model. The materials in the model are considered purely resistive due to the low frequency stimulation (20 Hz).
(DOCX)

S3 Table Biphasic stimulation with asymmetric charge-balanced (ACB) 1:4 waveforms directs full-length regeneration of crushed RGC axons. Average percent of regenerated RGCs at various distances from the crush site. Error represents SEM. SCB, symmetric charge-balanced.
(DOCX)

S4 Table Preserved retinal ganglion cell (RGC) viability with biphasic electrical stimulation. Wholemount retinas were processed for RBPMS immunohistochemistry. Baseline: 1 week after crush injury and electrode placement but before initiation of electric field (EF) stimulation. Mean RGC density +/- SEM. One-way ANOVA with Tukey’s multiple comparisons test. ACB, asymmetric charge-balanced; SCB, symmetric charge-balanced.
(DOCX)

S5 Table Summary table of subcortical visual targets with retinal ganglion cell (RGC) axon projections after asymmetric charge-balanced (ACB) 1:4 stimulation for 6 weeks. SCN = suprachiasmatic nucleus; vLGN = ventral lateral geniculate nucleus; IGL = intergeniculate leaflet; dLGN = dorsal lateral geniculate nucleus; OPN = olivary pretectal nucleus; NOT = nucleus of the optic tract; MPT = medial pretectal nucleus; PPT = posterior pretectal nucleus; SC = superior colliculus. N/A = damaged during surgery.—= no axons detected; + = rare axons detected; ++ = axons detected; +++ = abundant axons detected.
(DOCX)

S6 Table Biphasic stimulation with asymmetric charge-balanced (ACB) 1:4 waveforms mediates partial recovery of retinal ganglion cell function. Baseline: One week after crush and electrode placement but before initiation of stimulation. Mean normalized N95 amplitude on pattern electroretinography testing over time in each stimulation group (error bars, SEM). SCB, symmetric charge-balanced; UnTx, untreated.
(DOCX)

S7 Table Biphasic stimulation with asymmetric charge-balanced (ACB) 1:4 waveforms mediates partial recovery of local field potential recordings. Stereotactically guided local field potential (LFP) responses to full-field light stimulation were measured in the contralateral superior colliculus (SC). ∧ Only one animal with single response and thus no statistics could be calculated. One-way ANOVA with Dunnett’s multiple comparison test. * p < 0.05, ** p < 0.01. SCB, symmetric charge-balanced; UnTx, untreated.
(DOCX)

S8 Table Biphasic stimulation with asymmetric charge-balanced (ACB) waveforms failed to mediate recovery of visual behaviors. Rats stimulated with biphasic waveforms underwent visual cliff and optokinetic reflex (OKR) testing. SCB, symmetric charge-balanced; UnTx, untreated.
(DOCX)

S9 Table Biphasic stimulation with asymmetric charge-balanced (ACB) 1:4 waveforms for 2 weeks directs an intermediate level of regeneration of crushed retinal ganglion cell (RGC) axons compared with stimulation with ACB 1:4 waveforms for 6 weeks. Average percent of regenerated RGCs at various distances from the crush site. Error represents SEM. UnTx, untreated.
(DOCX)
