# Supplementary figures and images for: Electric field stimulation directs target-specific axon regeneration and partial restoration of vision after optic nerve crush injury
Source: PLoS One. 2025 Jan 9;20(1):e0315562. doi: 10.1371/journal.pone.0315562 (PMC11717274; doi:10.1371/journal.pone.0315562)

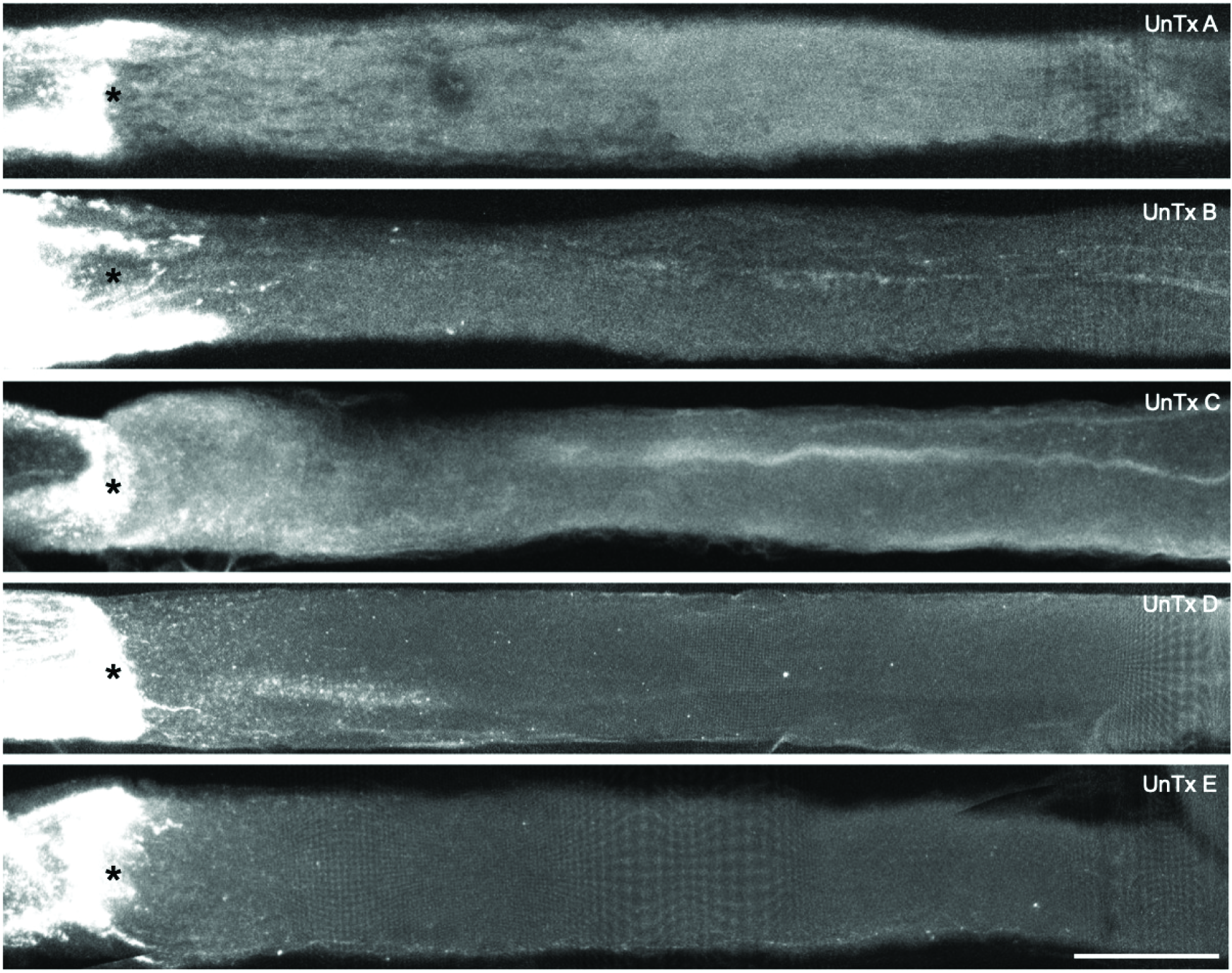

Supplement: S1 Fig — Orthogonal images (20x magnification) of cholera toxin B-labeled optic nerves of untreated animals. Few axons seen past the crush site (asterisk). Scale bar, 250 μm. UnTx, untreated. (TIF) [file pone.0315562.s001.tif]

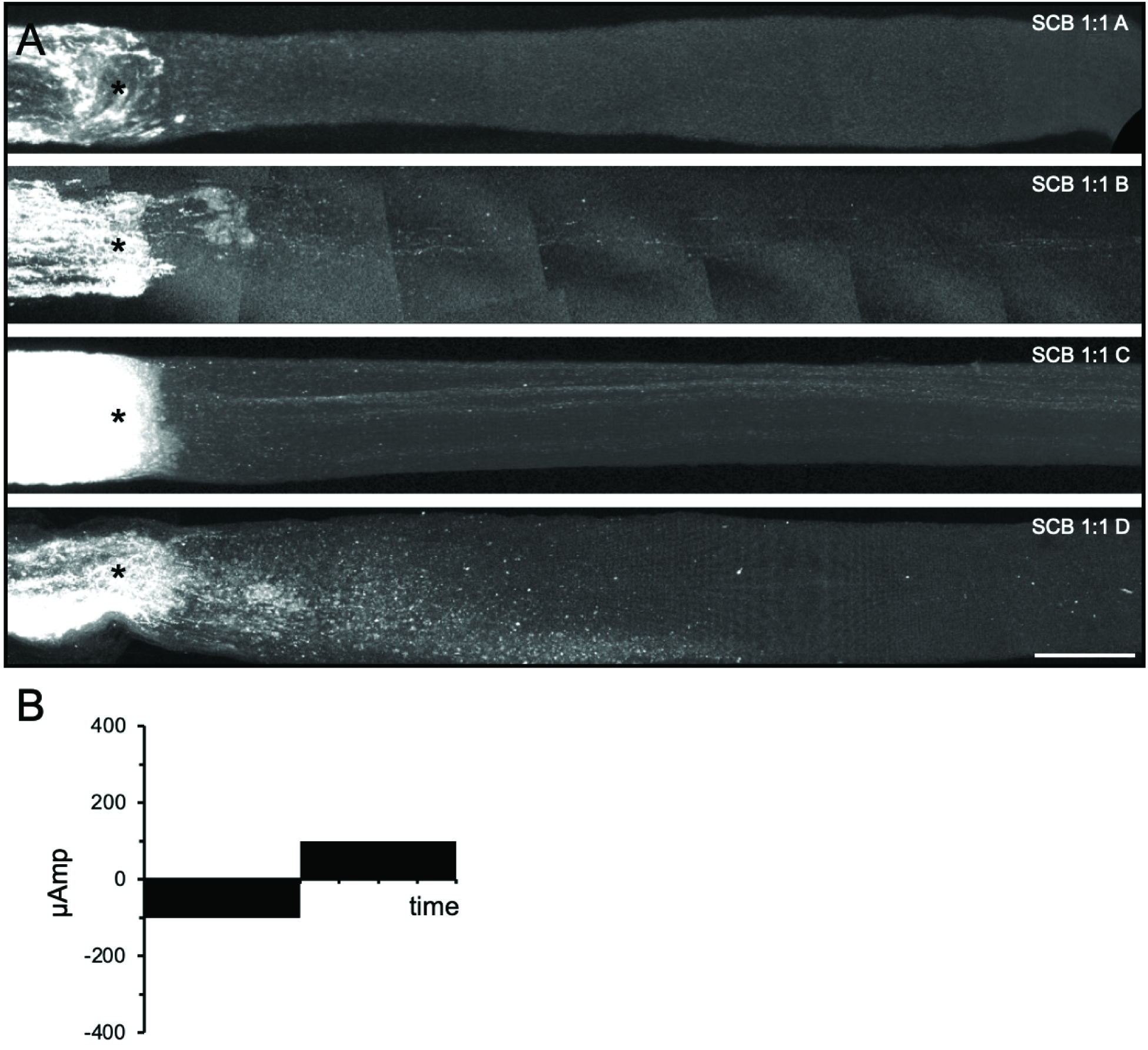

Supplement: S2 Fig — (A) Orthogonal images (20x magnification) of CTB-labeled optic nerves of animals in the SCB 1:1 group. Few axons seen past the crush site (asterisk). Scale bar, 250 μm. (B) Schematic of SCB 1:1 waveform. (TIF) [file pone.0315562.s002.tif]

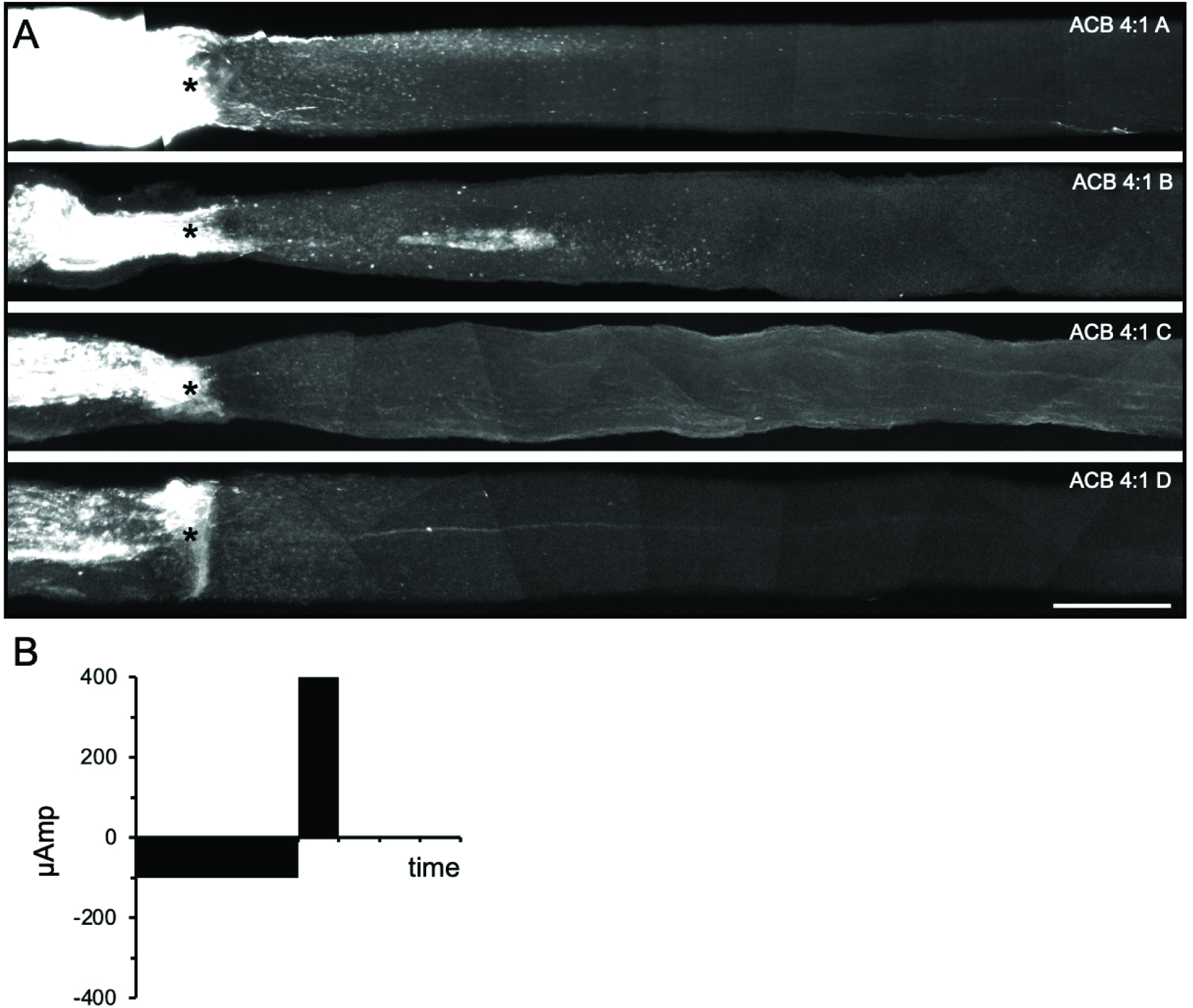

Supplement: S4 Fig — (A) Orthogonal images (20x magnification) of CTB-labeled optic nerves of animals in the ACB 4:1 group. Few axons seen past the crush site (asterisk). Scale bar, 250 μm. (B) Schematic of ACB 4:1 waveform. (TIF) [file pone.0315562.s004.tif]

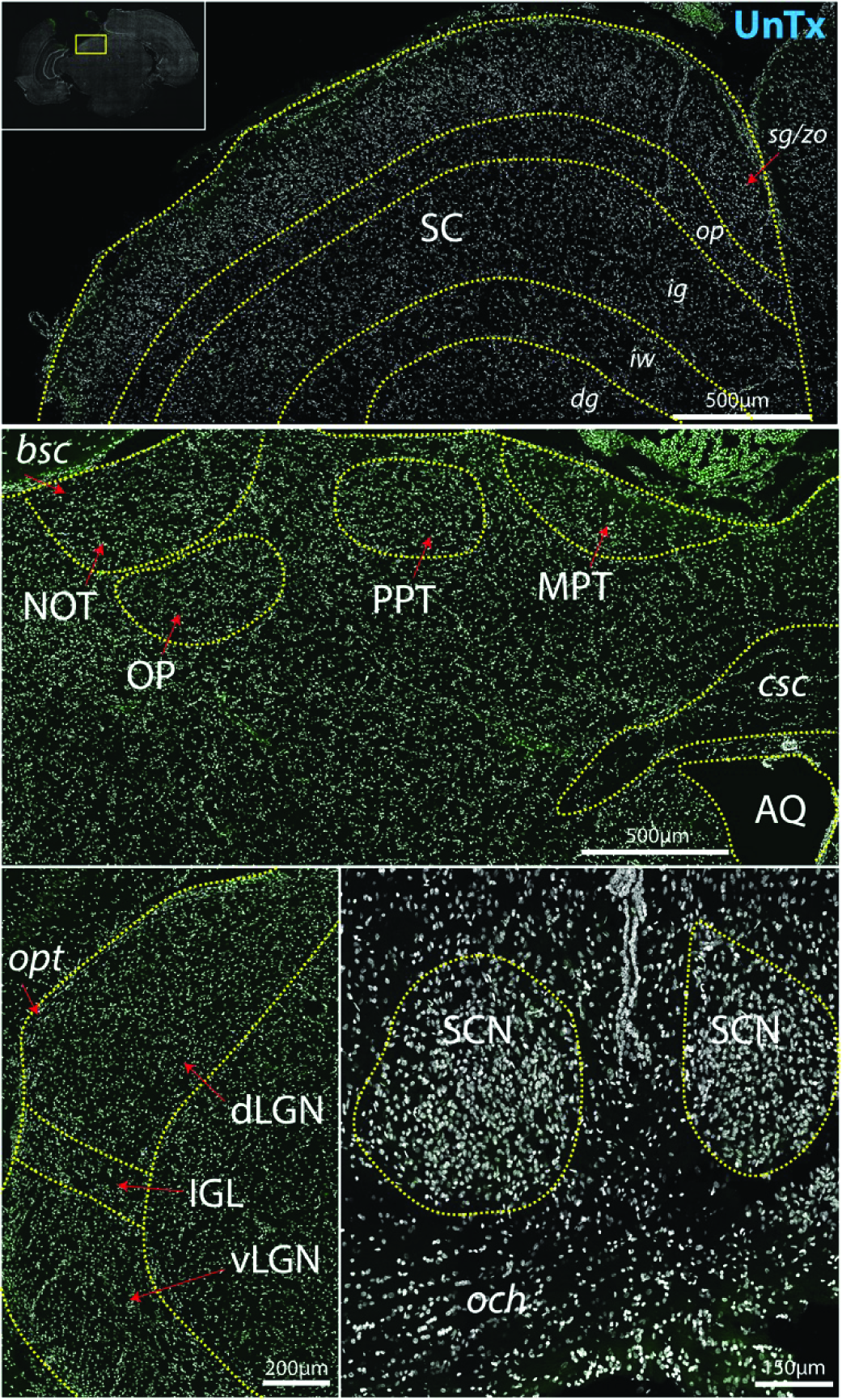

Supplement: S5 Fig — (A) In sham animals, no cholera toxin B (CTB)-labeled RGC axons can be seen projecting to subcortical visual targets, including bilateral SCN and contralateral vLGN, IGL, dLGN, OPN, NOT, MPT, PPT, and SC. SCN, suprachiasmatic nucleus; vLGN, ventral lateral geniculate nucleus; IGL, intergeniculate leaflet; dLGN, dorsal lateral geniculate nucleus; OPN, olivary pretectal nucleus; NOT, nucleus of the optic tract; MPT, medial pretectal nucleus; PPT, posterior pretectal nucleus; SC, superior colliculus. (TIF) [file pone.0315562.s005.tif]

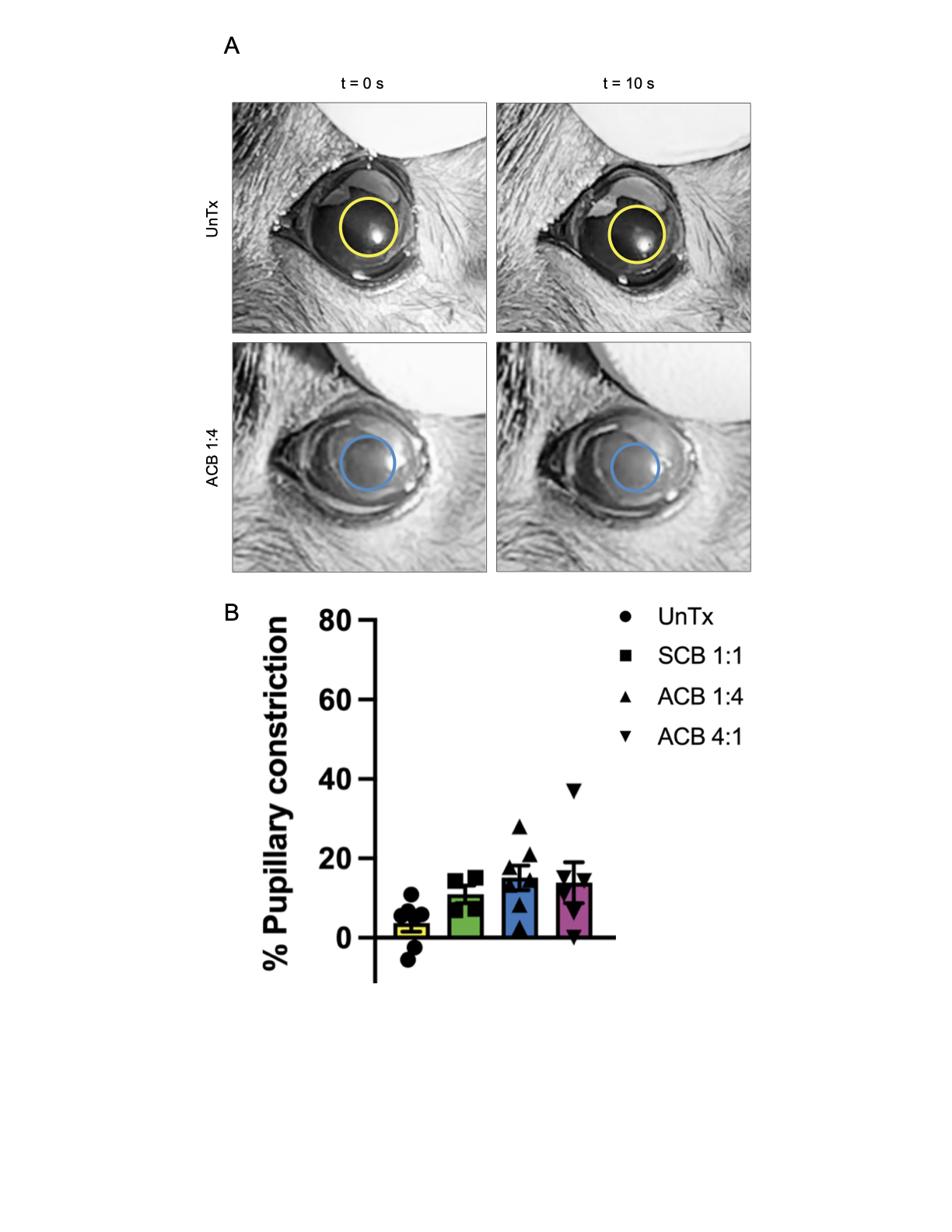

Supplement: S6 Fig — (A) Pupil circumference of an untreated animal (yellow circle) ACB 1:4 treated animal (blue circle) demonstrate no changed after 10 seconds of photic stimulation. (B) Quantification of pupillary change after 10 seconds of photic stimulation (UnTx, N = 7; SCB 1:1, N = 4; ACB 1:4, N = 7; ACB 4:1, N = 6; error bars, SEM; two-way ANOVA with Tukey’s multiple comparisons test). SCB, symmetric charge-balanced; UnTx, untreated. (TIFF) [file pone.0315562.s006.tiff]

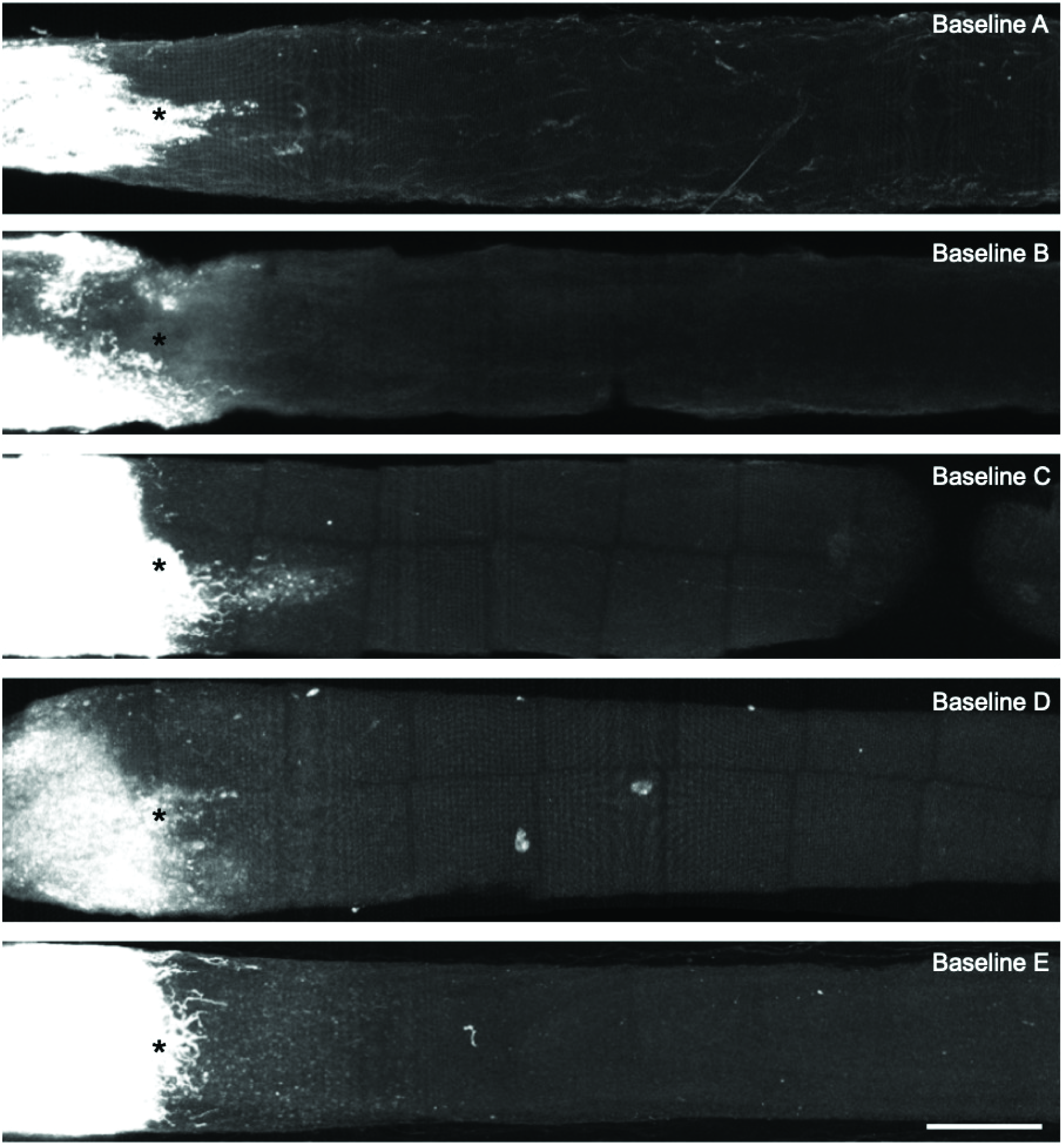

Supplement: S7 Fig — Orthogonal images (20x magnification) of cholera toxin B-labeled optic nerves of baseline animals. Few axons observed past the crush site (asterisk). Scale bar, 250 μm. (TIF) [file pone.0315562.s007.tif]

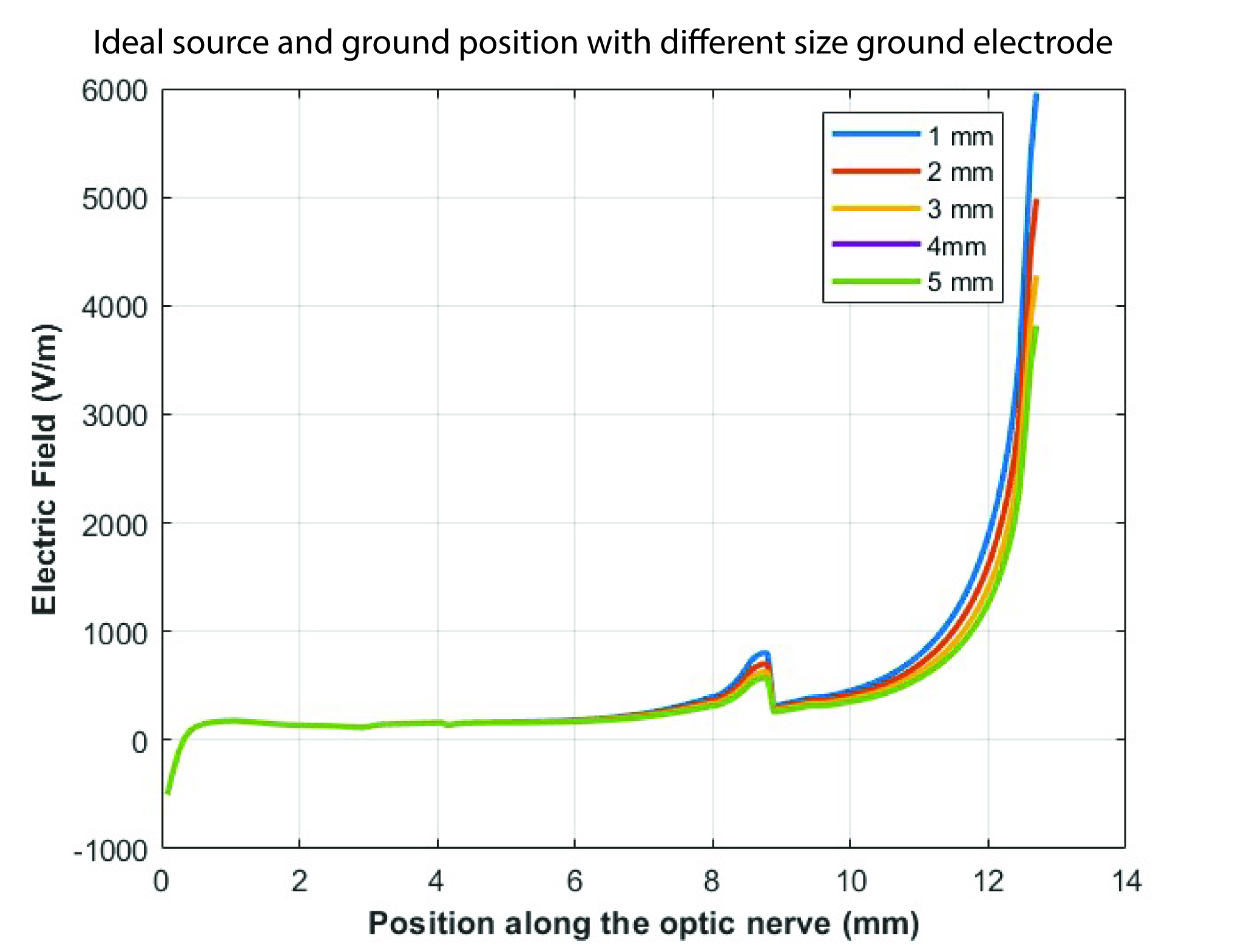

Supplement: S8 Fig — AM admittance method was used to generate a 3D computational model of the electric gradient along the optic nerve. Electric gradient along the optic nerve and tract with ideal source position (Condition 1, from Fig 7) and ideal ground position but with different size ground electrodes. (TIF) [file pone.0315562.s008.tif]
