# Supplementary material for: Electric field stimulation directs target-specific axon regeneration and partial restoration of vision after optic nerve crush injury
Source: PLoS One. 2025 Jan 9;20(1):e0315562. doi: 10.1371/journal.pone.0315562 (PMC11717274; doi:10.1371/journal.pone.0315562)
Supplement: S3 Fig — (A-I) Orthogonal images (20x magnification) of cholera toxin B-labeled optic nerves of animals in the ACB 1:4 waveform group. Many axons seen past the crush site (asterisk). (A1-I3) Select Z-stack images were collapsed and magnified from corresponding insets in (A-H) to reduce background noise and show the course of retinal ganglion cell (RGC) axons more clearly. Scale bars, 250 μm. (J) Schematic of ACB 1:4 waveform. (K) Quantification of RGC axon density at 250 μm intervals from the crush site after stimulation with various waveforms for 6 weeks (UnTx, N = 5; SCB 1:1, N = 4; ACB 1:4, N = 6; ACB 4:1, N = 4; error bars, SEM; * p < 0.05, ** p < 0.01, *** p < 0.001; two-way ANOVA with Tukey’s multiple comparisons test). (DOCX) [file pone.0315562.s003.docx]

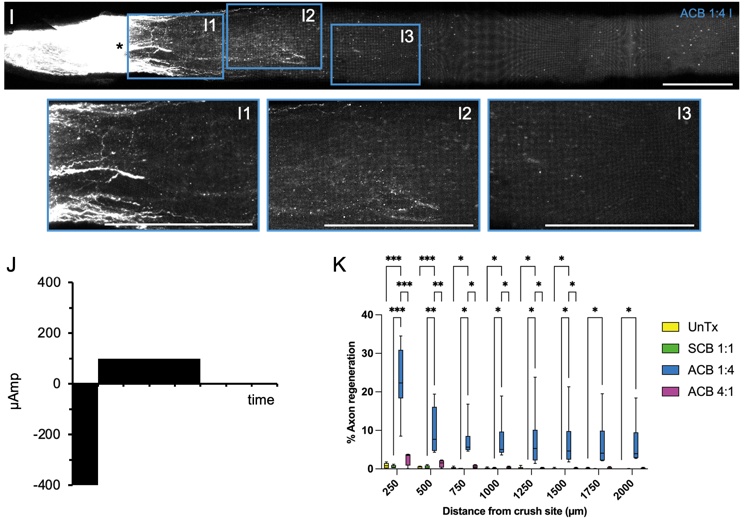

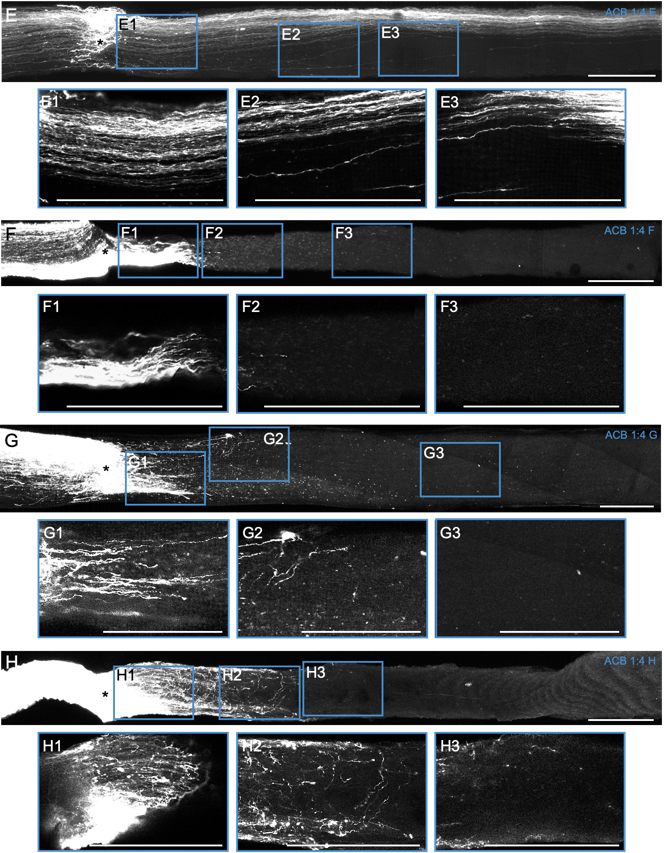

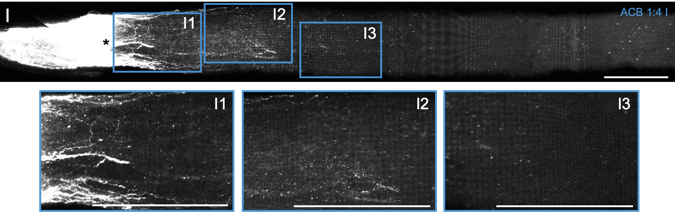

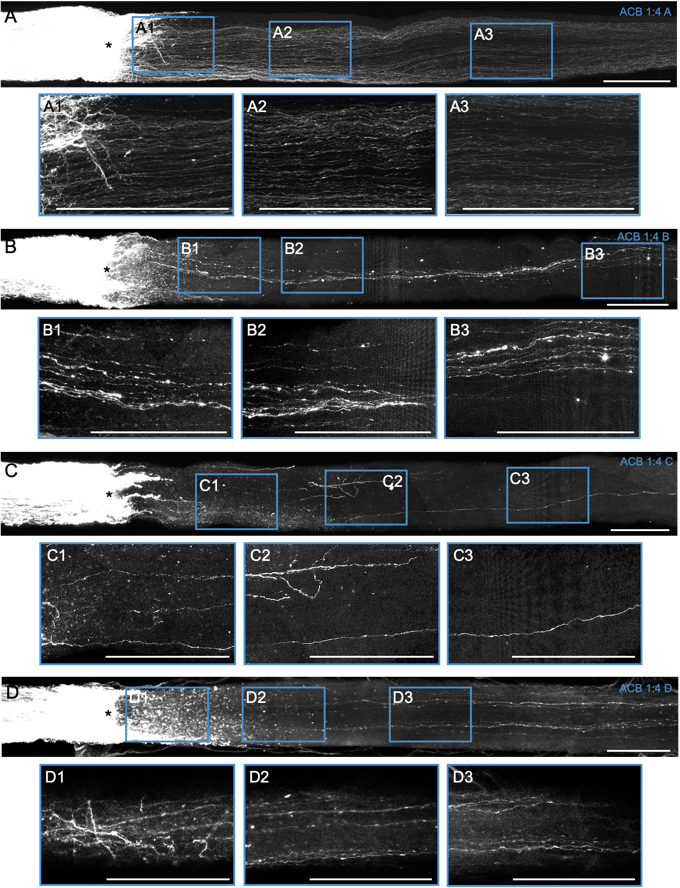

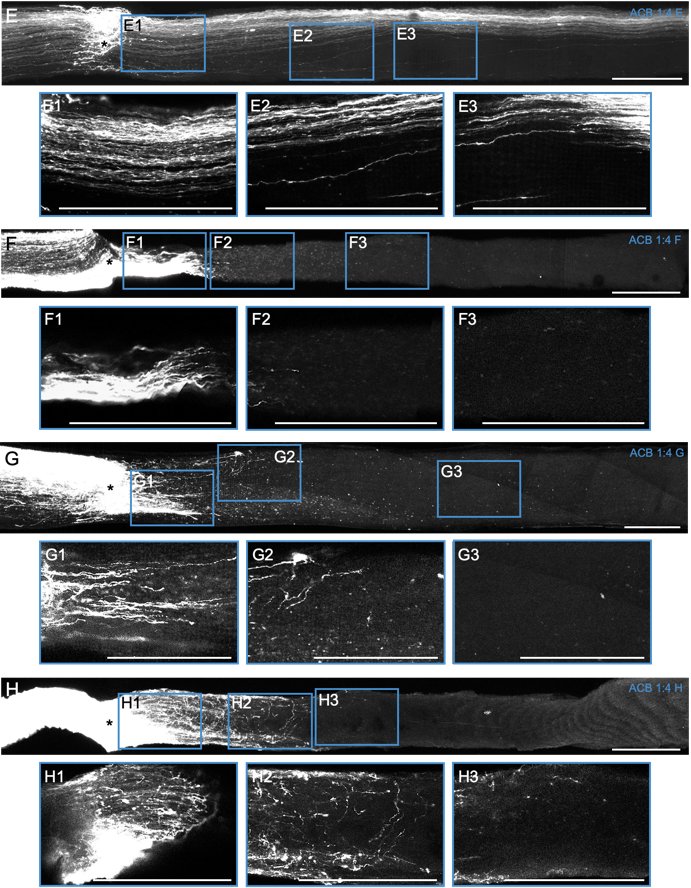


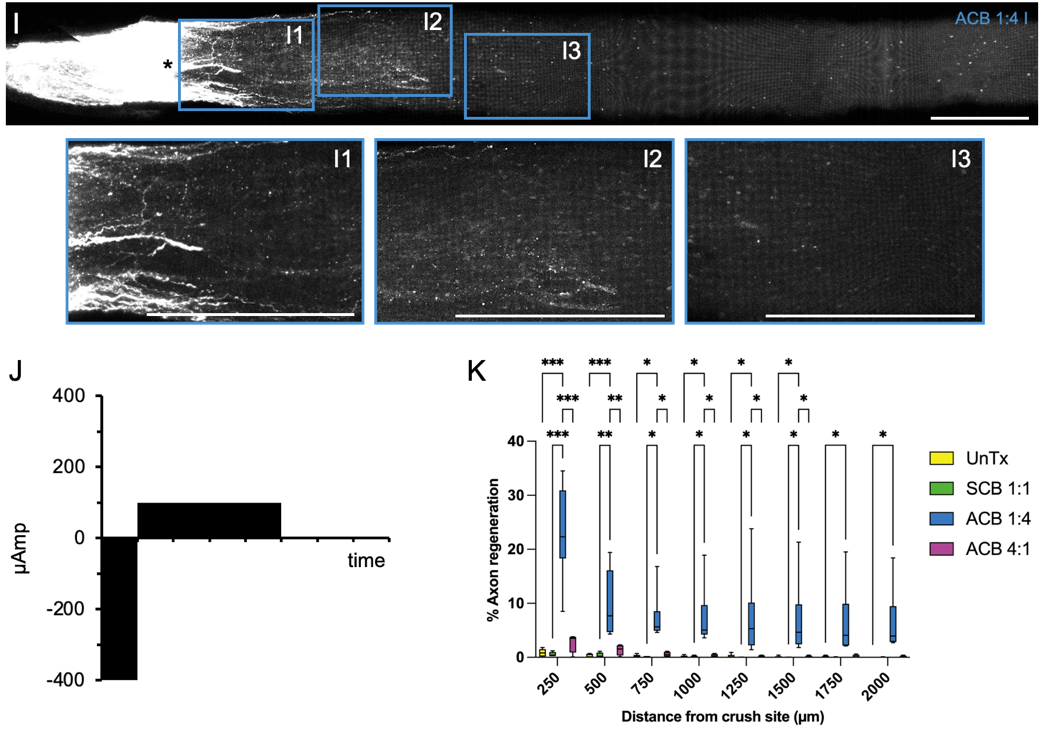


**Supplementary Figure 3. Optic nerves of animals treated with asymmetric charge-balanced (ACB) 1:4 waveforms for 6 weeks.** (A-I) Orthogonal images (20x magnification) of cholera toxin B-labeled optic nerves of animals in the ACB 1:4 waveform group. Many axons seen past the crush site (asterisk). (A1-I3) Select Z-stack images were collapsed and magnified from corresponding insets in (A-H) to reduce background noise and show the course of retinal ganglion cell (RGC) axons more clearly. Scale bars, 250 μm. (J) Schematic of ACB 1:4 waveform. (K) Quantification of RGC axon density at 250 μm intervals from the crush site after stimulation with various waveforms for 6 weeks (UnTx, N = 5; SCB 1:1, N = 4; ACB 1:4, N = 6; ACB 4:1, N = 4; error bars, SEM; * p < 0.05, ** p < 0.01, *** p < 0.001; two-way ANOVA with Tukey’s multiple comparisons test).
