# Supplementary material for: Electric field stimulation directs target-specific axon regeneration and partial restoration of vision after optic nerve crush injury
Source: PLoS One. 2025 Jan 9;20(1):e0315562. doi: 10.1371/journal.pone.0315562 (PMC11717274; doi:10.1371/journal.pone.0315562)
Supplement: S1 Table — Target stimulation parameters for each waveform. (DOCX) [file pone.0315562.s009.docx]

**Table S1: Target stimulation parameters.** Target stimulation parameters for each waveform.

| Waveform | Cathodic Amplitude (µAmp) | Anodic Amplitude (µAmp) | Cathodic Pulse Width (µsec) | Anodic Pulse Width (µsec) | Duty Cycle |
| --- | --- | --- | --- | --- | --- |
| SCB 1:1 | 100 | 100 | 400 | 400 | 50% |
| ACB 1:4 | 400 | 100 | 100 | 400 | 50% |
| ACB 4:1 | 100 | 400 | 400 | 100 | 50% |
