# Supplementary material for: Electric field stimulation directs target-specific axon regeneration and partial restoration of vision after optic nerve crush injury
Source: PLoS One. 2025 Jan 9;20(1):e0315562. doi: 10.1371/journal.pone.0315562 (PMC11717274; doi:10.1371/journal.pone.0315562)
Supplement: S2 Table — The materials in the model are considered purely resistive due to the low frequency stimulation (20 Hz). (DOCX) [file pone.0315562.s010.docx]

**Table S2: Resistivity of different materials used in the model.** The materials in the model are considered purely resistive due to the low frequency stimulation (20 Hz).

| Material | Resistivity | Material | Resistivity | Material | Resistivity |
| --- | --- | --- | --- | --- | --- |
| Skin Dry | 5000 | Deflated Lung | 4.93 | Wet Skin | 2456 |
| Muscle | 4.82 | Nerve | 42 | Lens | 3.15 |
| Fat | 60.16 | White Matter | 25.7 | Cornea | 2.4 |
| Bone Marrow | 762.95 | Grey Matter | 23.19 | Retina/Sclera | 1.99 |
| Heart | 16.13 | Cerebrospinal Fluid (CSF) | 0.5 | Vitreous | 44 |
