# Supplementary material for: Electric field stimulation directs target-specific axon regeneration and partial restoration of vision after optic nerve crush injury
Source: PLoS One. 2025 Jan 9;20(1):e0315562. doi: 10.1371/journal.pone.0315562 (PMC11717274; doi:10.1371/journal.pone.0315562)
Supplement: S3 Table — Average percent of regenerated RGCs at various distances from the crush site. Error represents SEM. SCB, symmetric charge-balanced. (DOCX) [file pone.0315562.s011.docx]

**Table S3: Biphasic stimulation with asymmetric charge-balanced (ACB) 1:4 waveforms directs full-length regeneration of crushed RGC axons.** Average percent of regenerated RGCs at various distances from the crush site. Error represents SEM. SCB, symmetric charge-balanced.

|  | N | 250 µm | 500 µm | 1000 µm | 2000 µm |
| --- | --- | --- | --- | --- | --- |
| UnTx | 5 | 0.82 +/- 0.32 | 0.36 +/- 0.15 | 0.14 +/- 0.09 | 0 +/- 0 |
| SCB 1:1 | 4 | 0.53 +/- 0.23 | 0.38 +/- 0.24 | 0.15 +/- 0.09 | 0.03 +/- 0.03 |
| ACB 1:4 | 6 | 23.15 +/- 3.61 | 9.82 +/- 2.50 | 7.27 +/- 2.36 | 6.40 +/- 2.46 |
| ACB 4:1 | 4 | 2.72 +/- 0.88 | 1.38 +/- 0.50 | 0.33 +/- 0.14 | 0.15 +/- 0.09 |
