# Supplementary material for: Electric field stimulation directs target-specific axon regeneration and partial restoration of vision after optic nerve crush injury
Source: PLoS One. 2025 Jan 9;20(1):e0315562. doi: 10.1371/journal.pone.0315562 (PMC11717274; doi:10.1371/journal.pone.0315562)
Supplement: S4 Table — Wholemount retinas were processed for RBPMS immunohistochemistry. Baseline: 1 week after crush injury and electrode placement but before initiation of electric field (EF) stimulation. Mean RGC density +/- SEM. One-way ANOVA with Tukey’s multiple comparisons test. ACB, asymmetric charge-balanced; SCB, symmetric charge-balanced. (DOCX) [file pone.0315562.s012.docx]

**Table S4: Preserved retinal ganglion cell (RGC) viability with biphasic electrical stimulation.** Wholemount retinas were processed for RBPMS immunohistochemistry. Baseline: 1 week after crush injury and electrode placement but before initiation of electric field (EF) stimulation. Mean RGC density +/- SEM.  One-way ANOVA with Tukey’s multiple comparisons test. ACB, asymmetric charge-balanced; SCB, symmetric charge-balanced.

|  | Group | N | Total area quantified, mm^2^ | # RBPMS + RGCs/0.5 mm^2^; +/-SEM |
| --- | --- | --- | --- | --- |
|  | Age matched control | 5 | 18.1 | 812 +/- 145 |
| 1 week post crush | Baseline | 3 | 3.27 | 468 +/- 66 |
| 2 weeks stimulation | UnTx | 3 | 13.5 | 27.3 +/- 11 |
|  | ACB 1:4 | 9 | 40.6 | 35.2 +/- 38 |
| 6 weeks stimulation | UnTx | 5 | 22.4 | 18.9 +/- 9 |
|  | SCB 1:1 | 4 | 18.1 | 19 +/- 14 |
|  | ACB 1:4 | 9 | 40.6 | 28.8 +/- 15 |
|  | ACB 4:1 | 5 | 22.6 | 15.5 +/- 6 |
