# Supplementary material for: Electric field stimulation directs target-specific axon regeneration and partial restoration of vision after optic nerve crush injury
Source: PLoS One. 2025 Jan 9;20(1):e0315562. doi: 10.1371/journal.pone.0315562 (PMC11717274; doi:10.1371/journal.pone.0315562)
Supplement: S5 Table — SCN = suprachiasmatic nucleus; vLGN = ventral lateral geniculate nucleus; IGL = intergeniculate leaflet; dLGN = dorsal lateral geniculate nucleus; OPN = olivary pretectal nucleus; NOT = nucleus of the optic tract; MPT = medial pretectal nucleus; PPT = posterior pretectal nucleus; SC = superior colliculus. N/A = damaged during surgery.—= no axons detected; + = rare axons detected; ++ = axons detected; +++ = abundant axons detected. (DOCX) [file pone.0315562.s013.docx]

**Table S5. Summary table of subcortical visual targets with retinal ganglion cell (RGC) axon projections after asymmetric charge-balanced (ACB) 1:4 stimulation for 6 weeks.** SCN = suprachiasmatic nucleus; vLGN = ventral lateral geniculate nucleus; IGL = intergeniculate leaflet; dLGN = dorsal lateral geniculate nucleus; OPN = olivary pretectal nucleus; NOT = nucleus of the optic tract; MPT = medial pretectal nucleus; PPT = posterior pretectal nucleus; SC = superior colliculus. N/A = damaged during surgery. - = no axons detected; + = rare axons detected; ++ = axons detected; +++ = abundant axons detected.

| Animal | SCN | vLGN | IGL | dLGN | OPN | NOT | MPT/PPT | SC |
| --- | --- | --- | --- | --- | --- | --- | --- | --- |
| ACB 1:4 A | N/A | ++ | + | ++ | + | ++ | + | +++ |
| ACB 1:4 B | - | ++ | + | ++ | + | + | + | + |
| ACB 1:4 C | + | - | - | - | - | - | + | + |
| ACB 1:4 D | N/A | + | + | + | - | ++ | + | ++ |
| ACB 1:4 E | + | +++ | + | +++ | ++ | +++ | ++ | +++ |
| ACB 1:4 F | + | - | - | - | - | - | - | - |
| ACB 1:4 G | - | - | - | - | - | - | - | - |
| ACB 1:4 H | - | - | - | - | - | - | - | - |
