# Supplementary material for: Electric field stimulation directs target-specific axon regeneration and partial restoration of vision after optic nerve crush injury
Source: PLoS One. 2025 Jan 9;20(1):e0315562. doi: 10.1371/journal.pone.0315562 (PMC11717274; doi:10.1371/journal.pone.0315562)
Supplement: S6 Table — Baseline: One week after crush and electrode placement but before initiation of stimulation. Mean normalized N95 amplitude on pattern electroretinography testing over time in each stimulation group (error bars, SEM). SCB, symmetric charge-balanced; UnTx, untreated. (DOCX) [file pone.0315562.s014.docx]

**Table S6. Biphasic stimulation with asymmetric charge-balanced (ACB) 1:4 waveforms mediates partial recovery of retinal ganglion cell function.** Baseline: One week after crush and electrode placement but before initiation of stimulation. Mean normalized N95 amplitude on pattern electroretinography testing over time in each stimulation group (error bars, SEM). SCB, symmetric charge-balanced; UnTx, untreated

| Group | N animals | Baseline | 2 weeks of stimulation | 6 weeks of stimulation |
| --- | --- | --- | --- | --- |
| UnTx | 5 | 0.45 +/- 0.02 | 0.33 +/- 0.07 | 0.18 +/- 0.03 |
| SCB 1:1 | 7 | 0.32 +/- 0.05 | 0.42 +/- 0.04 | 0.52 +/- 0.11 |
| ACB 1:4 | 8 | 0.37 +/- 0.04 | 0.49 +/- 0.06 | 0.68 +/- 0.07 |
| ACB 4:1 | 5 | 0.32 +/- 0.04 | 0.38 +/- 0.05 | 0.18 +/- 0.07 |
