# Supplementary material for: Electric field stimulation directs target-specific axon regeneration and partial restoration of vision after optic nerve crush injury
Source: PLoS One. 2025 Jan 9;20(1):e0315562. doi: 10.1371/journal.pone.0315562 (PMC11717274; doi:10.1371/journal.pone.0315562)
Supplement: S7 Table — Stereotactically guided local field potential (LFP) responses to full-field light stimulation were measured in the contralateral superior colliculus (SC). ∧ Only one animal with single response and thus no statistics could be calculated. One-way ANOVA with Dunnett’s multiple comparison test. * p < 0.05, ** p < 0.01. SCB, symmetric charge-balanced; UnTx, untreated. (DOCX) [file pone.0315562.s015.docx]

**Table S7**. **Biphasic stimulation with asymmetric charge-balanced (ACB) 1:4 waveforms mediates partial recovery of local field potential recordings.** Stereotactically guided local field potential (LFP) responses to full-field light stimulation were measured in the contralateral superior colliculus (SC). ^ Only one animal with single response and thus no statistics could be calculated. One-way ANOVA with Dunnett’s multiple comparison test. * p < 0.05, ** p < 0.01. SCB, symmetric charge-balanced; UnTx, untreated.

| Group | Total animals | Average # sites tested per animal | Average percent positive responses  SEM | # Animals with positive response | Average amplitude µV +/- STD | Average latency msec +/- STD |
| --- | --- | --- | --- | --- | --- | --- |
| Normal | 3 | 30.1 | 100% +/- 0% | 3 | 223.7 +/- 93.7 | 31.4 +/- 3.3 |
| Baseline | 5 | 27.8 | 1.25% +/- 1.25% | 1 | 93.4 ^ | 50.2 ^ |
| UnTx | 5 | 31 | 2.67 % +/- 1.64% | 2 | 76.6 +/- 71.8* | 93.6 +/- 2.9 |
| SCB 1:1 | 6 | 30.2 | 1.06% +/- 0.67% | 2 | 85.1 +/- 24.7* | 384.5 +/- 347.2** |
| ACB 1:4 | 8 | 31 | 20.69% +/- 4.01% | 8 | 81.8 +/- 18.1** | 113.6 +/- 31.4 |
| ACB 4:1 | 5 | 30.2 | 5.27% +/- 3.41% | 2 | 109.7 +/- 11.6 | 91.8 +/- 20.3 |
