# Supplementary material for: Electric field stimulation directs target-specific axon regeneration and partial restoration of vision after optic nerve crush injury
Source: PLoS One. 2025 Jan 9;20(1):e0315562. doi: 10.1371/journal.pone.0315562 (PMC11717274; doi:10.1371/journal.pone.0315562)
Supplement: S8 Table — Rats stimulated with biphasic waveforms underwent visual cliff and optokinetic reflex (OKR) testing. SCB, symmetric charge-balanced; UnTx, untreated. (DOCX) [file pone.0315562.s016.docx]

**Table S8: Biphasic stimulation with asymmetric charge-balanced (ACB) waveforms failed to mediate recovery of visual behaviors.** Rats stimulated with biphasic waveforms underwent visual cliff and optokinetic reflex (OKR) testing. SCB, symmetric charge-balanced; UnTx, untreated.

|  | N | % correct dismount to shallow side of visual cliff | N | % positive response to OKR |
| --- | --- | --- | --- | --- |
| UnTx | 5 | 40% | 5 | 0% |
| SCB 1:1 | 6 | 33.3% | 6 | 0% |
| ACB 1:4 | 8 | 25% | 8 | 0% |
| ACB 4:1 | 4 | 50% | 4 | 0% |
