# Supplementary material for: Electric field stimulation directs target-specific axon regeneration and partial restoration of vision after optic nerve crush injury
Source: PLoS One. 2025 Jan 9;20(1):e0315562. doi: 10.1371/journal.pone.0315562 (PMC11717274; doi:10.1371/journal.pone.0315562)
Supplement: S9 Table — Average percent of regenerated RGCs at various distances from the crush site. Error represents SEM. UnTx, untreated. (DOCX) [file pone.0315562.s017.docx]

**Table S9: Biphasic stimulation with asymmetric charge-balanced (ACB) 1:4 waveforms for 2 weeks directs an intermediate level of regeneration of crushed retinal ganglion cell (RGC) axons compared with stimulation with ACB 1:4 waveforms for 6 weeks.** Average percent of regenerated RGCs at various distances from the crush site. Error represents SEM. UnTx, untreated/

|  | N | 250 µm | 500 µm | 750 µm | 1000 µm |
| --- | --- | --- | --- | --- | --- |
| Baseline | 5 | 0.34 +/- 0.24 | 0.02 +/- 0.02 | 0 +/- 0 | 0 +/- 0 |
| UnTx 2 Weeks | 6 | 0.48 +/- 0.25 | 0.15 +/- 0.11 | 0 +/- 0 | 0 +/- 0 |
| ACB 1:4 2 Weeks | 8 | 6.96 +/- 1.94 | 2.26 +/- 0.88 | 1.18 +/- 0.42 | 1.09 +/- 0.48 |
| ACB 1:4 6 Weeks | 6 | 23.15 +/- 3.61 | 9.82 +/- 2.50 | 7.25 +/- 1.92 | 7.27 +/- 2.36 |
